# Mahogunin regulates fusion between amphisomes/MVBs and lysosomes via ubiquitination of TSG101

**DOI:** 10.1038/cddis.2015.257

**Published:** 2015-11-05

**Authors:** P Majumder, O Chakrabarti

**Affiliations:** 1 Biophysics and Structural Genomics Division, Saha Institute of Nuclear Physics, Sector 1, Block-AF, Bidhannagar, Kolkata 700064, India

## Abstract

Aberrant metabolic forms of the prion protein (PrP), membrane-associated ^Ctm^PrP and cytosolic (cyPrP) interact with the cytosolic ubiquitin E3 ligase, Mahogunin Ring Finger-1 (MGRN1) and affect lysosomes. MGRN1 also interacts with and ubiquitinates TSG101, an ESCRT-I protein, involved in endocytosis. We report that MGRN1 modulates macroautophagy. In cultured cells, functional depletion of MGRN1 or overexpression of ^Ctm^PrP and cyPrP blocks autophagosome–lysosome fusion, alleviates the autophagic flux and its degradative competence. Concurrently, the degradation of cargo from the endo-lysosomal pathway is also affected. This is significant because catalytic inactivation of MGRN1 alleviates fusion of lysosomes with either autophagosomes (via amphisomes) or late endosomes (either direct or mediated through amphisomes), without drastically perturbing maturation of late endosomes, generation of amphisomes or lysosomal proteolytic activity. The compromised lysosomal fusion events are rescued by overexpression of TSG101 and/or its monoubiquitination in the presence of MGRN1. Thus, for the first time we elucidate that MGRN1 simultaneously modulates both autophagy and heterophagy via ubiquitin-mediated post-translational modification of TSG101.

All cells rely on efficient lysosomal degradation for maintenance of their homoeostasis, perturbations in this leads to several debilitating diseases. Lysosomes are specialized organelles that degrade macromolecules received from the secretory, endocytic, autophagic and phagocytic pathways. Autophagy is considered as a ubiquitous bulk degradation mechanism of damaged organelles and long lived, misfolded or accumulated proteins.^1^ Activated growth factors, hormones, cytokine receptors, misfolded plasma membrane proteins are internalized by endocytosis and delivered to the lysosomes via the multivesicular bodies (MVBs), a mechanism also termed as heterophagy. Interestingly defects in either of the pathways have been associated with the pathogenesis of numerous neurodegenerative diseases.^2^

Perturbations in autophagy-related protein (ATG) genes, *Atg7* and *Atg5* lead to developmental defects during organogenesis^3, 4^ or even neonatal death.^5^ Similarly, studies have reported that null mutations in the lysosomal membrane protein LAMP2 result in general myopathy and cardiomyopathy.^6, 7^ Lysosomal degradation is essential for normal physiological activity in neurons. Anomalies at various stages in the maturation of the endosomes through MVBs to lysosomes or during the *de novo* generation of autophagosomes result in neurodegenerative diseases like Alzheimer's disease and Huntington's disease.^8, 9^

Many other neurodegenerative diseases like Parkinson's disease, Niemann–Pick type C disease, frontotemporal dementia (FTD) and amyotropic lateral sclerois (ALS) are also referred as ‘lysosomal diseases'. These are all associated with dysfunction of the ESCRT (endosomal sorting complex required for transport) machinery, comprising a pathway of five distinct complexes (ESCRTs -0, -I, -II and -III, and Vps4), which recognize and sort ubiquitinated cargo through an exquisite division of labor.^10^ Depletion or mutations in the molecular players of the ESCRT complexes severely affects the structure and function of endo-lysosomal compartments.^11, 12, 13, 14^ These proteins also facilitate autophagy by affecting fusion events involving lysosomes, endosomes and autophagosomes.^15, 16, 17, 18, 19, 20^

In context of this, it is worth indicating that loss of *Mgrn1* (Mahogunin Ring Finger-1) function leads to late-onset spongiform neurodegeneration in selected brain regions, very similar to prion disease pathology.^21^ Catalytically MGRN1, a cytosolic ubiquitin E3 ligase is implicated in lysosomal dysfunction.^22, 23^ MGRN1 can interact with a transmembrane prion protein (PrP) isoform (^Ctm^PrP), associated with familial or inherited disease.^23^ It is also suggested to be involved in the clearance of cytosolic chaperone heat shock 70 kDa protein (HSP70)-associated misfolded proteins.^24^ Although it is prudent to suggest that MGRN1 could have a role in certain familial prion diseases, recent evidence does not indicate its involvement in transmissible spongiform encephalopathy.^25^ However, this does not undermine the role of MGRN1 in regulating lysosomal degradation.

Here, we dissect the mechanism by which MGRN1 regulates lysosomal degradation. We have identified a novel role MGRN1 in modulating autophagy. Depletion of MGRN1 disrupts both amphisomal–lysosomal and endo-lysosomal degradation pathways. These effects are due to the blocked fusion of vesicles with lysosomes and can be rescued by overexpression of TSG101 and/or its monoubiquitination. MGRN1 can modulate clearance of cargo at the lysosomes by regulating vesicular fusion events.

## Results

### MGRN1 affects macroautophagy

**Figure 1:**
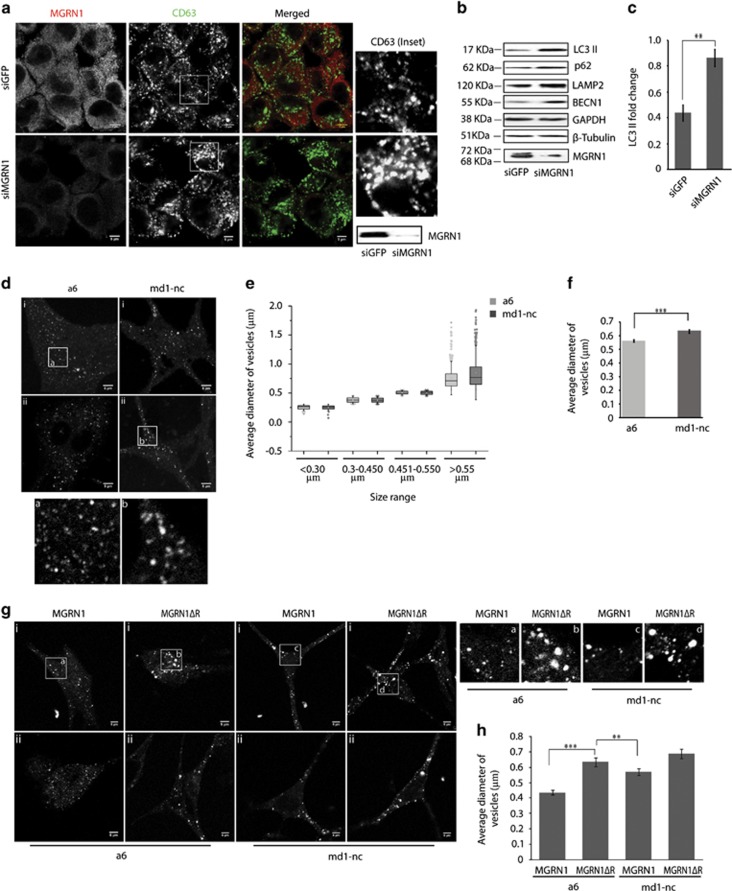
Compromised function of MGRN1 affects markers of late endocytosis/lysosomes and autophagy. (**a**) HeLa cells treated with irrelevant siRNAs (GFP siRNAs) or MGRN1 siRNAs, co-immunostained for CD63 and MGRN1 were imaged. Note a qualitative increase in size of vesicles with the functional depletion of MGRN1. The channels for acquiring the images are indicated. Enlarged views of the areas within the white boxes are also shown (insets). Scale bar, 5 *μ*m. The immunoblot on the right shows efficiency of siRNA-mediated MGRN1 knockdown. (**b**) HeLa cells similarly treated as in (**a**) were lysed and immunoblotted for autophagy and lysosomal proteins. The levels of GAPDH and *β-*tubulin serve as loading controls. The blots are representative of at least three experiments. Efficiency of knockdown was checked using anti-MGRN1 antibody. Note that the antibody used against LC3, detects only endogenous LC3 II. (**c**) The immunoblots shown in (**b**) were analyzed for the levels of LC3 II. Graph shows results from three independent experiments. **P≤0.05, using Student's *t*-test. Error bars, ±S.E.M. (**d**) Melanocytes, melan a6 and melan md1-nc cells were stained with lysotracker and imaged. Lack of MGRN1 causes enlargement of acidic vesicles. Two fields for each cell line are shown. Insets a (melan a6) and b (melan md1-nc) reveal enlarged views of the vesicles. Scale bar, 5 *μ*m. (**e**) Graph shows the size distribution of lysotracker positive vesicles imaged in (**d**). Over 1000 vesicles from at least 25 cells are represented for each cell line. The horizontal line in each box shows the median value and the white square inside each box is the mean. The upper and lower boundaries of individual box show the upper and lower quartiles, the whiskers are S.D. Outlier values are shown outside the whiskers. (**f**) The average diameter of vesicles analyzed in (**e**) shows a minor but significant increase in size in melan md1-nc cells as compared with the control, melan a6 cells. ***P≤0.001, using Student's *t*-test. Error bars, ±S.E.M. (**g**) MGRN1 or MGRN1ΔR was ectopically expressed in melan a6 and melan md1-nc cells. The samples were stained with lysotracker and imaged. Enlarged views of the areas within the white boxes (insets) are also shown. Scale bar, 5 *μ*m. (**h**) The effect of MGRN1 or MGRN1ΔR overexpression in melanocytes was plotted (mean±S.E.M.) for ~100 vesicles. Note that the overexpression of MGRN1 partially rescues the enlarged lysosomal/vesicular phenotype, while MGRN1ΔR aggravates it. **P≤0.05, ***P≤0.001 using Student's *t-*test. Error bars, ±S.E.M.

Depletion of MGRN1 function in HeLa and SHSY5Y cells altered the morphology of late endosomes and/or lysosomes (Figure 1a and Supplementary Figure S1A), similar to earlier reports.^22, 23^ The physiologic reason for this phenotype, however, has remained elusive. MGRN1 depletion resulted in increased LAMP2 protein levels. Also, similarly affected were autophagy proteins, like, Beclin1 (BECN1), LC3 II and p62 (Figures 1b and c, Supplementary Figures S1B and S1F) – implying aberrant autophagy-mediated lysosomal degradation. As an indirect support for this hypothesis, we checked the status of autophagy proteins in ^Ctm^PrP or cyPrP containing cell lysates. These PrP isoforms are suggested to interact with and partially phenocopy MGRN1 depletion.^23^ Several constructs known to generate enhanced levels of ^Ctm^PrP were used.^26, 27, 28^ HuPrP(A117V) expression led to decrease in the ubiquitination activity of MGRN1 (Supplementary Figure S2). Elevated levels of GFP-LC3 II were observed in HeLa cells co-transfected with GFP-LC3 and the different PrP constructs known to generate increased amounts of ^Ctm^PrP or cyPrP (Supplementary Figure S1C). It was logical to assume that alteration of MGRN1 function had an important role in regulating macroautophagy.

### Lack of MGRN1 affects acidic vesicles (late endosomes/lysosomes)

Next, we checked if MGRN1 deficiency had an effect on macroautophagy and lysosomes. Marginal but significant increase in size of acidic vesicles, as visualized with lysotracker staining, was detected in melanocytes derived from *Mgrn1* null mice,^29^ melan md1-nc (Figures 1d–f) compared with melan a6 (control) cells. To validate the involvement of MGRN1 in the lysosomal phenotype, both melanocytes were transfected with full length MGRN1 or catalytically inactive RING deleted MGRN1ΔR (Figures 1g and h). Overexpression of MGRN1 in melan md1-nc cells partially rescued the phenotype. MGRN1ΔR expression in melan a6 cells resulted in substantially larger lysosomes, while MGRN1 reduced this. These suggested a role of MGRN1 in lysosomal alterations. Surprisingly, the protein levels of LC3 II were significantly low in the melan md1-nc cells, compared with the control, melan a6 cells (Supplementary Figures S1D and S1E). The p62 levels were, however, similar in both the cell lines. It seemed justified that if these cells were to survive and propagate in culture for multiple passages (over 30), high levels of LC3 II (signifying abnormal autophagy) could be detrimental for the cells. A compensatory mechanism is probably responsible for this regulation. p62 is a multipurpose protein.^30^ Hence, maintaining its basal levels would be essential for stable homoeostasis and propagation of melan md1-nc cell lines. However, it cannot be completely ruled out that the discrepancies observed in the LC3 II and p62 levels between siRNA-treated and *Mgrn1* null cells-derived samples could be attributed to incomplete knockdown and partial residual activity of MGRN1.

### MGRN1 depletion leads to blocked fusion of autophagosomes with late endosomes and/or lysosomes

Enlarged size of late endosomes and/or lysosomes, accompanied with increased levels of autophagy and lysosomal proteins, raised two possibilities. First, accumulation of cargo brought in via macroautophagy at the lysosomes without its efficient degradation. Second, in a completely opposite scenario, if the rate of autophagy/proteolysis in lysosomes was high, it would demand increased synthesis of vesicle-associated proteins and hence result in their elevated levels. In various cell types, depletion of MGRN1 led to an increase in the number and average size of RFP-LC3 vesicles (autophagosomes), hence strengthening the possibility of cargo accumulation without efficient degradation (Supplementary Figures S3A–S3F).

**Figure 2:**
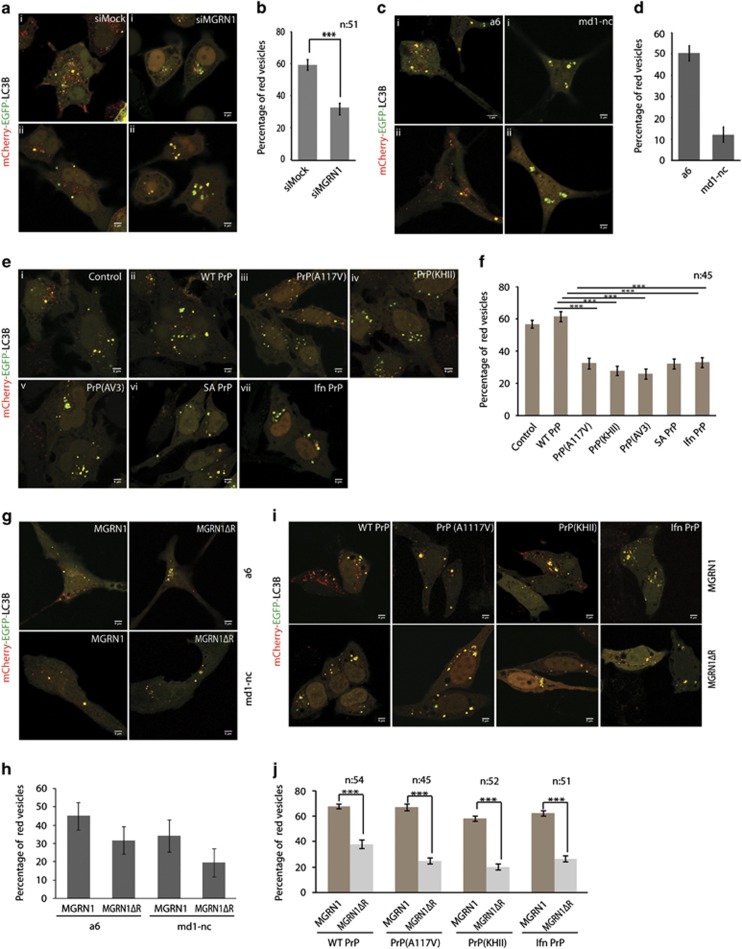
Altered processing of dual–tagged LC3B when MGRN1 function is either reduced or absent. (**a**) HeLa cells treated with irrelevant siRNAs (mock siRNAs) or MGRN1 siRNAs, were transfected with mCherry-EGFP-LC3B construct. 72h post transfection, cells were fixed and imaged. Yellow and red vesicles indicate autophagosomes and autolysosomes, respectively. Two fields for each condition are shown. Scale bar, 5 *μ*m. (**b**) Graph shows the percentage of red vesicles among total vesicles for cells imaged in (**a**). The number of fluorescent bodies per cell among ~51 cells was calculated from three independent experiments (over 30 fields). It shows a significant decrease (~2 fold) in the percentage of red vesicles (calculated out of the total (yellow±red) vesicles for the entire sample set) using the given equation: percentage of red vesicles=(average number of red vesicles in *n* cells/average number of (yellow±red) vesicles) × 100. ***P-value≤0.001 using Student's *t*-test. Error bars, ±S.E.M. (**c**) Melan a6 and melan md1-nc cells were transfected with mCherry-EGFP-LC3B and imaged 24 h later. Note that total absence of MGRN1 (as in melan md1-nc cells) closely phenocopies the effects of its partial depletion in HeLa. Scale bar, 5 *μ*m. Two fields for each cell line are shown. (**d**) Histogram was generated using the same sample set from (**c**). Histogram shows ~4.1-folds decrease in percentage of red vesicles in melan md1-nc cells, compared with the control melan a6 cells. Ten cells (total number of vesicles, ~250) were analyzed. Error bars, ±S.E.M. (**e**) HeLa cells transiently co-transfected with various PrP constructs and mCherry-EGFP-LC3B were imaged 24 h later. Scale bar, 5 *μ*m. (**f**) Histogram plotting the percentage of red vesicles in PrP expressing HeLa cells. Approximately 45 cells were analyzed for each PrP construct. The percentage of red vesicles in the empty vector control or upon overexpression of wild-type PrP was ~57% or 62% of the total number of vesicles, respectively. This number varied between 26 and 32% for all PrP constructs known to increase the amounts of ^Ctm^PrP generated (that included artificial constructs PrP(AV3), PrP(KH-II) and SA-PrP, as well as the naturally occurring human disease mutation PrP(A117V)). In the presence of Ifn-PrP (which generated enhanced amounts of cyPrP), there were ~33% red vesicles. ***P≤0.001, using Student's *t*-test. Error bars, ±S.E.M. (**g**) Melan a6 and melan md1-nc cells co-transfected with mCherry-EGFP-LC3B and either functional MGRN1 or inactive MGRN1ΔR were imaged. Two fields for each cell line are shown. Scale bar, 5 *μ*m. (**h**) Graph with results from (**g**) showing percentage of red vesicles for indicated constructs. Overexpression of MGRN1 in melan md1-nc cells, partially rescued and elevated the percentage of red vesicles to ~39% (that is >3 folds of untransfected cells in Figure 2d). MGRN1ΔR expression could not, however, aggravate the phenotype already exhibited in the untransfected melan md1-nc cells. In melan a6 cells, overexpression of MGRN1 did not drastically affect the percentage of red vesicles. On the contrary, exogenous MGRN1ΔR expression in them affected vesicular fusion with the lysosomes and resulted in a decrease in the percentage of red vesicles. Approximately 10 cells were analyzed. Histogram indicates that overexpression of MGRN1 and MGRN1ΔR exhibit opposite effects. Error bars, ±S.E.M. (**i**) HeLa cells co-transfected with the indicated PrP constructs, mCherry-EGFP-LC3B and either functional MGRN1 or inactive MGRN1ΔR were imaged. Scale bar, 5 *μ*m. (**j**) Images taken for each PrP construct were analyzed and plotted for the percentage of red vesicles among total vesicles. The graph indicates that overexpression of MGRN1 partially rescues altered processing of mCherry-EGFP-LC3B, while MGRN1ΔR aggravates this phenomenon. Number of cells analyzed for each PrP construct are indicated. *** *P*≤0.001, using Student's *t*-test. Error bars, ±S.E.M.

Presence of functionally compromised MGRN1 in cell culture systems led to significant decrease in the percentage of red vesicles, as monitored by the expression of dual-tagged LC3B^31^ (mCherry-EGFP-LC3B, with pH labile GFP and acid-stable mCherry) (Figures 2a and b and Supplementary Figures S3G–S3J). SHSY5Y cells expressing MGRN1ΔR and subjected to neuronal differentiation with all-*trans*-retinoic acid (RA) treatment^32^ also showed lower abundance of red vesicles as against those with MGRN1 (Supplementary Figure S3K). This number was also lower in *Mgrn1* null cells as against the melan a6 cells (Figures 2c and d). Hence, functional inactivation of MGRN1 (complete or partial) compromised the fusion of autophagosomes (pH neutral vesicles) with late acidic vesicles (endosomes and/or lysosomes), in a cell line-independent manner. It is worth noting that melan md1-nc cells showed a decrease in the percentage of acidic vesicles indicative of reduced lysosomal fusion, despite a lack of increase in levels of LC3 II (Supplementary Figure S1D).

Expression of various ^Ctm^PrP or cyPrP generating constructs led to at least two-folds decrease in the percentage of red vesicles compared with the controls (having empty vector or wild-type PrP) (Figures 2e and f).

In all the cases where MGRN1 function was compromised, it was observed that the total number of all vesicles (yellow and red) also decreased with a simultaneous increase in size.

Further, we checked whether exogenous expression of MGRN1 could mitigate aberrant lysosomal fusion (Figures 2g and h). Overexpression of MGRN1 in melan md1-nc cells partially rescued the phenotype and increased the percentage of red vesicles. MGRN1ΔR expression could not, however, aggravate the phenotype beyond the untransfected melan md1-nc cells (Figures 2g and h and Supplementary Figure S3L). In melan a6 cells, while overexpression of MGRN1 had no drastic effect, exogenous MGRN1ΔR expression resulted in a decrease in the percentage of red vesicles. Similarly, ^Ctm^PrP or cyPrP-mediated perturbation of lysosomal fusion could be rescued by the overexpression of MGRN1 (Figures 2i and j). Unlike in melan md1-nc cells, where MGRN1ΔR did not alleviate the percentage of red vesicles, overexpression of catalytically inactive MGRN1 in the presence of ^Ctm^PrP or cyPrP further reduced this percentage.

Blocking lysosome-mediated degradation by bafilomycin A1 (vacuolar H±ATPase inhibitor) is known to enrich LC3B in neutral vesicles with size comparable to acidic compartments.^31^ Drug-treated control HeLa cells (with MGRN1 or PrP) showed accumulation of neutral yellow vesicles in similar size range as the acidic red vesicles of the untreated controls (Supplementary Figures S3M and S3N). Bafilomycin A1 treatment did not have any drastic morphological impact on the already enlarged yellow vesicles after perturbation of MGRN1 function (by expressing MGRN1ΔR or PrP(A117V)).

Catalytically compromised MGRN1 hence altered the fusion of autophagosomes with late endosomes and/or lysosomes, also affecting efficient degradation of cargo at the lysosomes.

### Blocked fusion affects autophagic flux

**Figure 3:**
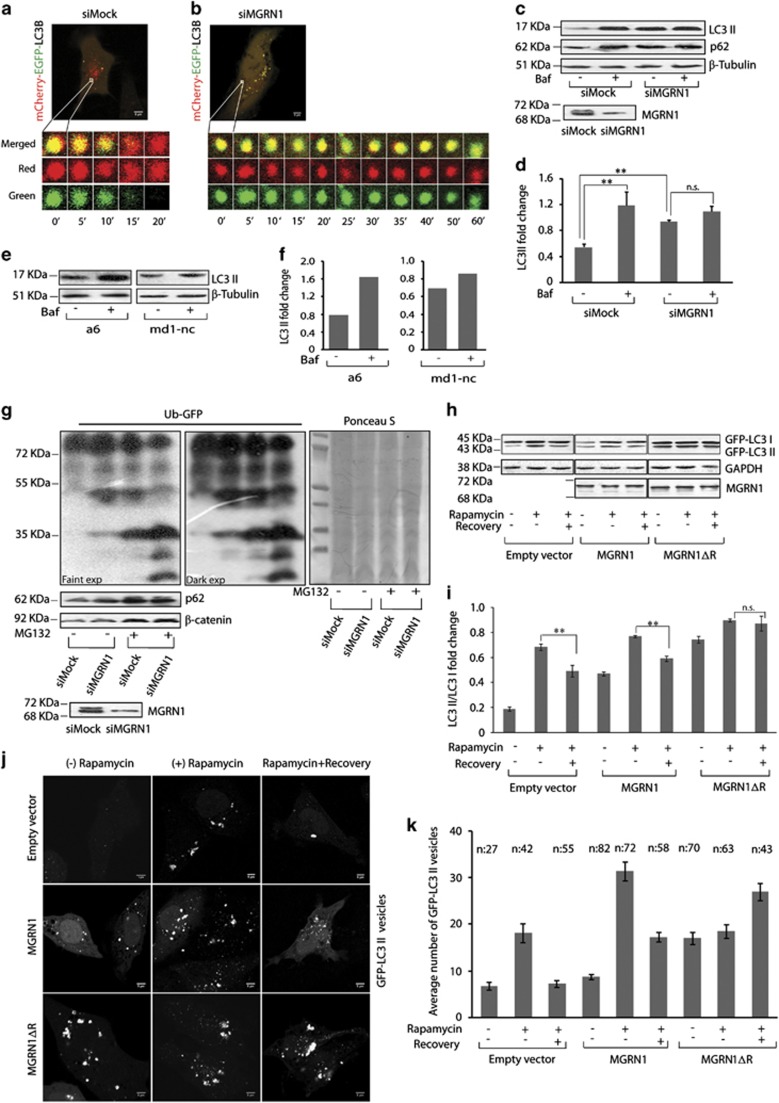
Compromise in MGRN1 activity perturbs autophagic fusion events. (**a**) HeLa cells treated with irrelevant siRNAs (mock siRNAs) were transfected with mCherry-EGFP-LC3B construct; live cells were imaged over indicated time periods to track the fate of vesicles in real time. One representative field, out of 25 cells is shown. Scale bar, 5 *μ*m. (**b**) Cells treated with MGRN1 siRNAs and transfected with mCherry-EGFP-LC3B construct were imaged similar to (**a**). One representative field, out of 25 cells is shown. Scale bar, 5 *μ*m. (**c**) Mock or MGRN1 siRNA-transfected HeLa cells were lysed and immunoblotted to analyze the levels of endogenous LC3 II and p62 in the presence or absence of 300 nM bafilomycin A1. The levels of *β-*tubulin serve as loading control. Efficiency of knockdown was confirmed by immunoblotting with anti-MGRN1. The blots are representative of at least three experiments. (**d**) The immunoblots from (**c**) were analyzed for the levels of LC3 II. Graph shows fold change in LC3 II when normalized against corresponding *β-*tubulin in the cell lysates, analyzed from three independent experiments. **P≤0.05, n.s., not significant (*P*=0.18) using Student's *t*-test. Error bars, ±S.E.M. (**e**) Melan a6 and melan md1-nc cells either treated with 50 nM bafilomycin A1 (for 4 h) or left untreated, were lysed and immunoblotted to check for the indicated proteins. This is representative of two independent experiments. (**f**) Quantification of data from (**e**) denotes fold change in endogenous LC3 II level when normalized against *β-*tubulin. (**g**) HeLa cells treated with mock or MGRN1 siRNAs were transiently transfected with Ub^G76V^-GFP construct (denoted as Ub-GFP). In the last 6 h before harvesting, they were grown in the presence or absence of 10 *μ*M MG132. Cell lysates from these samples were immunoblotted with the indicated antibodies. Faint and dark exposures show accumulation of Ub-GFP with MGRN1 depletion. Ponceaus-S stained membrane shows equal loading of total protein across samples. RNAi efficiency was shown using MGRN1 antibody. (**h**) HeLa cells transiently co-transfected with GFP-LC3 and empty vector, MGRN1 or MGRN1ΔR were either left untreated or treated with rapamycin for 24 h. Cells transfected with empty vector and GFP-LC3 were used as controls. Following this, cells were either immediately lysed or allowed to recover from the drug treatment for 12 h and then harvested. All samples were immunoblotted using anti-GFP and anti-MGRN1 antibodies. GAPDH was used as loading control. The blots are representative of at least three experiments. (**i**) Histogram shows fold change in the ratio of GFP-LC3 II/I when normalized against GAPDH from (**h**). Graph was generated from three independent experiments. **P≤0.05, n.s., not significant (*P*=0.67) using Student's *t*-test. Error bars, ±S.E.M. (**j**) Cells treated similar to (**h**) were imaged for the presence of green fluorescent structures. Scale bar, 5 *μ*m. (**k**) Cells imaged in (**j**) were scored for total number of GFP-LC3 positive vesicles and average number of such vesicles was calculated. *n*, total number of cells for the various treatments are indicated in the figure. Error bars, ±S.E.M.

Live cells imaged to monitor vesicle fusion in real time showed that a single yellow mCherry-EGFP-LC3B vesicle turned completely red over a course of ~20–30 min (Figure 3a and Supplementary Figure S4A).^33^ MGRN1 knockdown led to prolonged retention of GFP signal (Figure 3b and Supplementary Figures S4B–S4D), suggesting impeded fusion of autophagosomes with acidic vesicles. It was hence plausible to extrapolate that MGRN1 depletion would affect the autophagic flux.

Inhibiting lysosomal acidification with bafilomycin A1 treatment should block LC3 and p62 degradation.^33^ In control cells, this drug treatment significantly increased endogenous LC3 II levels compared with the untreated samples (Figures 3c and d and Supplementary Figures S5A, S5B and S5C). Functionally inactive MGRN1 did not elicit a similar response, rather elevated LC3 II levels were detected even without addition of bafilomycin A. This confirmed that compromised MGRN1 activity indeed blocked autophagic flux. Exogenously expressed GFP-LC3 II levels changed similarly as the endogenous LC3 II in HeLa cells when MGRN1 or MGRN1ΔR were present (Supplementary Figure S5B). Results were also corroborated in SHSY5Y cells (Supplementary Figures S5D and S5E). Bafilomycin A1 treatment increased LC3 II levels in melan a6 cells (Figures 3e and f), however, it did not educe comparable changes in the *Mgrn1* null melanocytes. Further, MGRN1 depletion in melan a6 cells, led to increased LC3 II levels; bafilomycin A1 treatment again did not yield a discernable difference (Supplementary Figures S5F and S5G). Thus emphasizing that the effect of MGRN1 on autophagic flux was cell line independent – while partial functional loss of MGRN1 elicited an increase in LC3 II, total depletion reduced the basal protein levels. In either case bafilomycin A1 treatment did not induce any further alteration in LC3 II levels.

Compromising autophagy is known to affect flux through the ubiquitin-proteasome system (UPS) as has been demonstrated previously using an artificial reporter – Ub^G76V^-GFP. It accumulates when proteasome is impaired,^34^ but may also do so when autophagy is blocked.^35^ MGRN1 knockdown led to an increase in protein levels of Ub^G76V^-GFP (Figure 3g), like during deficiencies of *Atg7*, *Atg12* or *Atg5*.^35^ p62 levels were also elevated as before (Figures 1b and 3g); however, *β*-catenin (a proteasomal substrate) remained unaltered. MG132 treatment increased p62 and *β*-catenin levels, irrespective of the functionality of MGRN1. Accumulation of Ub^G76V^-GFP in the presence of the proteasome inhibitor MG132 but not the autophagy blocker bafilomycin A1 helped ascertain its validity as a substrate for the UPS (Supplementary Figure S5H).

To measure the autophagy degradative competence, cells were treated with rapamycin and assayed for clearance of LC3 before and after drug withdrawal. Rapamycin treatment led to elevated GFP-LC3 II levels. After drug withdrawal, a significant reduction in this protein was detected in controls contrary to unvarying higher levels in MGRN1ΔR cells (Figures 3h and i). A complementary effect was also seen in imaging studies. The number of GFP-LC3 increased vesicles upon rapamycin treatment irrespective of the status of MGRN1. However, drug withdrawal had remarkably different effects – in MGRN1ΔR cells, the total number of vesicles remained high as the treated sample, while their numbers decreased in controls (Figures 3j and k).

MGRN1 activity hence regulated autophagic flux and its degradative competence.

### MGRN1 affects fusion of vesicles with lysosomes

**Figure 4:**
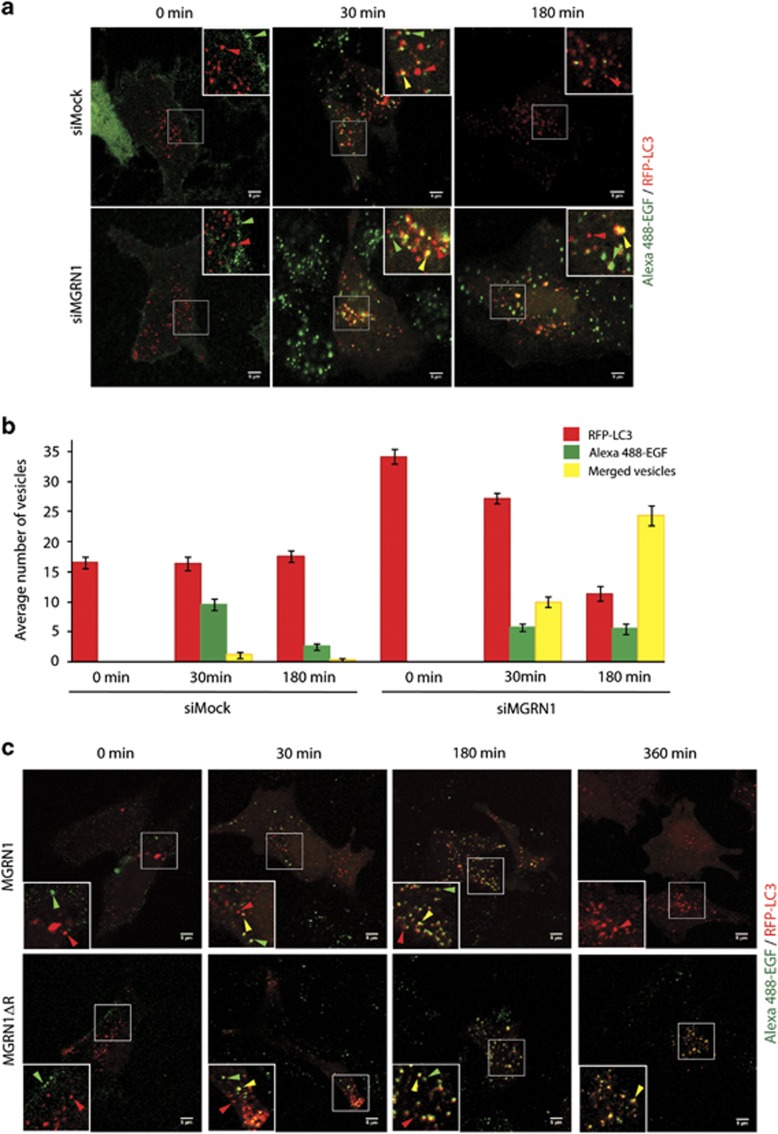
Functional aberration of MGRN1 blocks fusion of vesicles with lysosomes. (**a**) HeLa cells treated with mock or MGRN1 siRNAs and transiently transfected with RFP-LC3 construct were subjected to Alexa-Fluor 488 EGF uptake. Cells were washed, fixed at indicated time points and imaged. Insets reveal enlarged views of green (Alexa-Fluor 488-labeled EGF), red (RFP-LC3) and yellow (Alexa-Fluor 488 EGF and RFP-LC3 colocalized) vesicles as shown by respective colored arrow heads. Scale bar, 5 *μ*m. (**b**) Graph represents average number of red, green and yellow fluorescent vesicles analyzed over five fields. Error bars, ±S.E.M. (**c**) SHSY5Y cells transiently transfected with MGRN1 or MGRNΔR and RFP-LC3 construct were subjected to Alexa-Fluor 488 EGF uptake in a similar experiment as (**a**). The time points at which fixed cells were imaged are indicated. Insets reveal enlarged views of green (Alexa-Fluor 488-labeled EGF), red (RFP-LC3) and yellow (Alexa-Fluor 488 EGF and RFP-LC3 colocalized) vesicles as shown by respective colored arrow heads. Scale bar, 5 *μ*m

MGRN1 affects EGFR degradation and its downstream signaling.^22^ Our results re-iterated that epidermal growth factor (EGF)-induced EGFR degradation and clearance required functional MGRN1 (Supplementary Figure S6). It was, prudent to postulate that MGRN1 modulated lysosomal degradation by stalling one/more of the vesicular fusion events.^20^ To distinguish between the different pathways, mock or MGRN1 knockdown HeLa cells were transfected with RFP-LC3 and monitored for EGF uptake and degradation over a period of time. Samples had comparable presence of RFP-LC3 vesicles and loading with Alexa-Fluor 488-labeled EGF on the cell surface (Figures 4a and b). After 30 min of chase, both the cell populations had green (Alexa-Fluor 488-labeled EGF-EGFR internalized endosomes), red (RFP-LC3 autophagosomes) and yellow (amphisomes) vesicles. At 180 min, green, red and yellow vesicles could be detected in MGRN1-depleted cells; control cells retained only red punta. Occurrence of yellow vesicles could be extrapolated to suggest that MGRN1 depletion did not affect amphisome formation. Similar sequence of events was observed in SHSY5Y cells, except with slower kinetics (Figure 4c).

All these pointed to defective fusion of vesicles with the lysosome when MGRN1 was functionally inactive.

### MGRN1 does not perturb lysosomal competence

**Figure 5:**
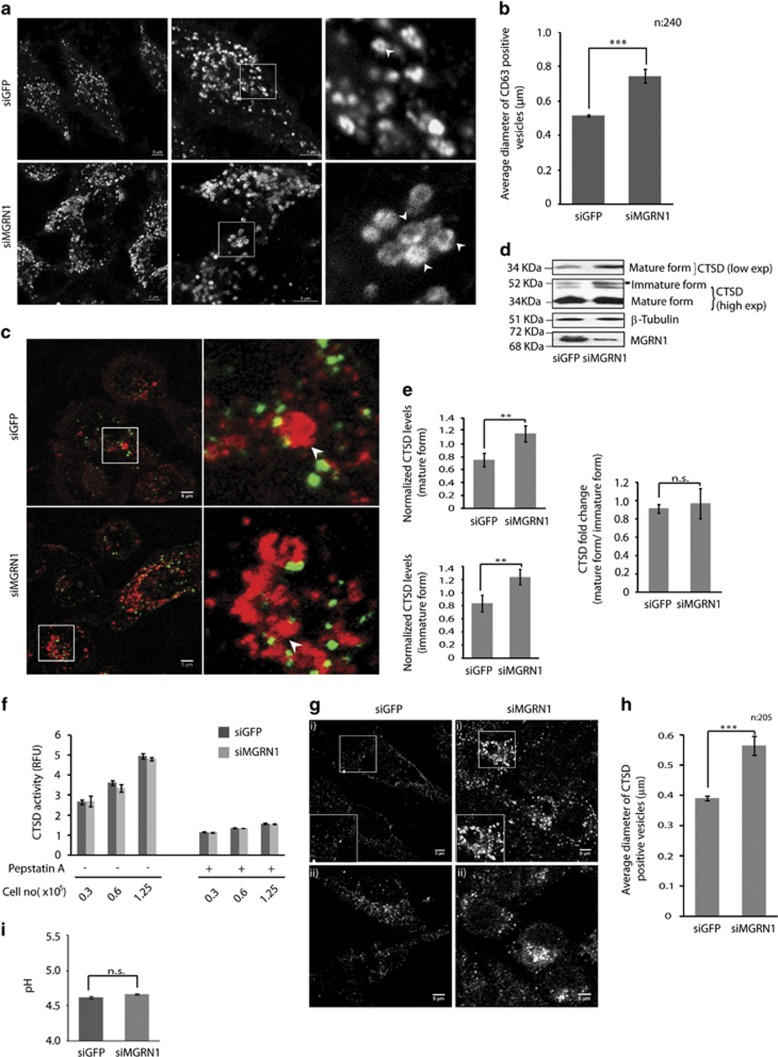
MGRN1 does not affect lysosomal competence. (**a**) HeLa cells treated with irrelevant (GFP) or MGRN1 siRNAs were fixed and immunostained with anti-CD63 antibody. Enlarged views of the areas within the white boxes (insets) are also shown. Scale bar, 5 *μ*m. (**b**) Graph plotting data from (**a**) shows significant increase (~1.5 folds) in average diameter of CD63 positive vesicles for MGRN1-depleted cells, where the vesicles diameter are ~0.78 *μ*m and diameter of vesicles in control cells are ~0.51 *μ*m. Note the presence of intra-luminal vesicles in both the samples, except that the ones with MGRN1 depletion are bigger in size. Approximately 240 vesicles were counted from three independent experiments. ***P≤0.001, using Student's *t*-test. Error bars, ±S.E.M. (**c**) HeLa cells treated with irrelevant (GFP) or MGRN1 siRNAs subjected to Alexa-Fluor 488 EGF uptake. Cells were washed, fixed at 40 min time point, immunostained for CD63 and imaged. Enlarged views of the areas within the white boxes (insets) are also shown. Insets reveal enlarged views of multiple Alexa-Fluor 488-labeled EGF positive puncta (green) on CD63 positive vesicles with intra-luminal membranes (red). Scale bar, 5 *μ*m. (**d**) Cell lysates treated with indicated siRNAs were analyzed for the levels of CTSD. The levels of *β-*tubulin serve as loading control. The low and dark exposures of the CTSD blot indicate the different processed forms of the enzyme. Efficiency of knockdown was confirmed by immunoblotting with anti-MGRN1. The blots are representative of at least three experiments. (**e**) Histograms plotting data from (**d**) show significant (~1.5 folds) but similar increase in the levels of mature and immature CTSD, as normalized against the protein levels of *β-*tubulin (top left) and (bottom left) with the depletion of MGRN1. However, note that the fold change in the mature and immature forms of CTSD are comparable between control and MGRN1 siRNA-treated samples (right). Graph representing three independent experiments. **P≤0.05, n.s., not significant (*P*=0.75) using Student's *t*-test. Error bars, ±S.E.M. (**f**) Cell lysates were analyzed for CTSD activity. Histogram plotted for the activity of this enzyme in MGRN1 and GFP siRNA-treated samples. To block CTSD activity, cells were either treated with vehicle control or pepstatin A, as indicated. Graph represents average of three independent experiments, performed in triplicate for each cell concentration. Error bars, ±S.E.M.; RFU, relative fluorescence units. (**g**) HeLa cells treated with irrelevant (GFP) or MGRN1 siRNAs were fixed and immunostained with anti-CTSD antibody. Two fields for each condition are shown. (**h**) Graph plotting the average diameter of vesicles showed significant increase (~1.4 folds) when MGRN1 is depleted, imaged in (**g**). Approximately 205 vesicles were counted from three independent experiments. ***P≤0.001, using Student's *t*-test. Error bars, ±S.E.M. (**i**) Lysosomal pH values were measured ratiometrically using LysoSensor yellow/blue DND-160–Dextran. In control cells, the average lysosomal pH was detected as 4.62±0.02, while in cells treated with MGRN1 siRNAs the pH was 4.66±0.01; n.s., not significant (*P*=0.9), using Student's *t*-test. Graph represents average of three independent experiments. Error bars, ±S.E.M.

The data so far did not rule out the possibilities of compromised formation of MVBs or impaired lysosomal acidification and/or proteolysis. Immunocytochemistry revealed the presence of CD63 positive vesicles with intra-luminal membranes in control and MGRN1-depleted HeLa cells (Figure 5a). Compromised MGRN1 activity resulted in significantly larger vesicles (Figure 5b). CD63 positive Alexa-Fluor 488-labeled EGF-EGFR internalized MVBs were detected across all samples (Figure 5c). Thus MGRN1 activity did not steer formation or maturation of the MVBs. Next, the competence of the lysosomes was evaluated. Approximately 1.5-folds increased levels of the resident aspartic endoprotease, Cathepsin D (CTSD) were detected in MGRN1-depleted samples (Figures 5d and e). However, the ratio between the pro-enzyme and mature forms was similar to the controls (Figure 5e, right graph). Pro-CTSD is targeted to endosomes and lysosomes;^36, 37^ their acidic environment helps generate the mature forms.^38^ Proper proteolytic cleavage of CTSD signified that the enzyme had reached its destination compartment where ambient pH is optimum for its processing. As this processing also involves other lysosomal cysteine proteases,^39^ it justified to state that MGRN1 did not alter lysosomal function. CTSD activity was comparable between control and MGRN1 depleted samples, effect of pepstatin A was also similar (Figure 5f). Hence, a rational extrapolation would be that a portion of CTSD accumulated in an endocytic compartment – thereby causing an increase in its protein levels without proportional alteration in activity. Cells with MGRN1 siRNAs had significantly enlarged CTSD positive vesicles, morphologically similar to the previously described CD63 compartments, suggesting that a fraction of these compartments might be late endosomes/MVBs (Figures 5g and h). Further, fluorimetric measurement of average lysosomal pH by LysoSensor Yellow/Blue showed insignificant change between control (4.62±0.02) and MGRN1-depleted samples (4.66±0.01) (Figure 5i). These results were corroborated using the same pH-sensitive dye in confocal experiments (Supplementary Figure S7). These results imply that the physiological nature of the late endosomes/MVBs or lysosomes is unperturbed.

### Lysosomal fusion with vesicles requires functional TSG101

**Figure 6:**
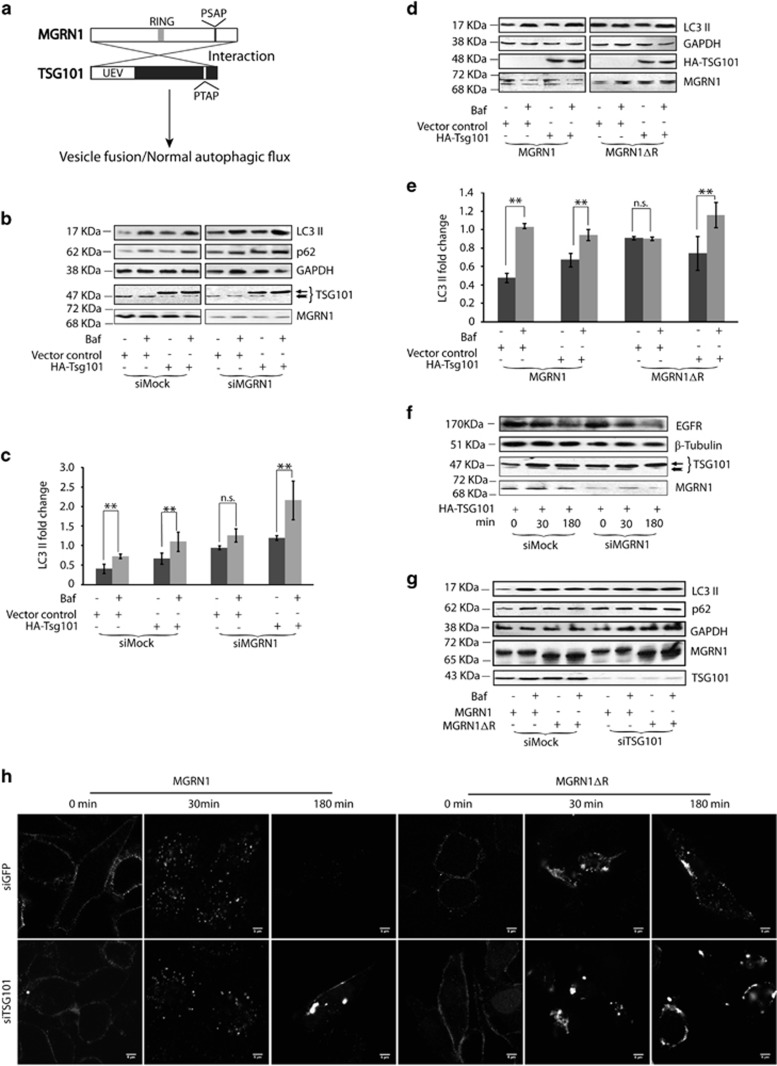
Autophagy and endo-lysosomal pathway defects caused by MGRN1 are mediated via TSG101. (**a**) Experimental logic to establish that MGRN1 interacts with TSG101 to mediate vesicular fusion with lysosomes. (**b**) HeLa cells treated with the indicated siRNAs were transfected with either empty vector or HA-TSG101. Cell lysates were immunoblotted to analyze the levels of endogenous LC3 II and p62 in the presence or absence of 300 nM bafilomycin A1. GAPDH was used as loading control. Efficiency of knockdown was shown by immunoblotting with anti-MGRN1. 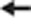 indicates HA-TSG101; 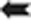 endogenous TSG101. The blots are representative of at least three independent experiments. (**c**) Graph shows fold change in LC3 II when normalized against corresponding GAPDH levels; analyzed from three independent experiments. **P≤0.05, n.s., not significant, (*P*=0.2) using Student's *t*-test. Error bars, ±S.E.M. (**d**) SHSY5Y cells co-transfected with MGRN1 or MGRNΔR construct and either empty vector or HA-TSG101. Cell lysates were immunoblotted to analyze the levels of endogenous LC3 II in the presence or absence of 60 nM bafilomycin A1. GAPDH was used as loading control. Efficiency of all transfections was checked. The blots are representative of at least three independent experiments. (**e**) Graph shows fold change in LC3 II when normalized against corresponding GAPDH levels; analyzed from three independent experiments. **P≤0.05, n.s., not significant, (*P*=0.8) using Student's *t*-test. Error bars, ±S.E.M. (**f**) Cells treated with the indicated siRNAs and transfected with HA-TSG101 were subjected to EGF uptake. Lysates were analyzed for the levels of EGFR at specified time intervals. *β*-tubulin was used as loading control. 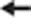 indicates HA-TSG101; 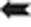 endogenous TSG101. Efficiencies of knockdown and transfection were also checked. (**g**) In a reverse experiment, HeLa cells were treated with mock or TSG101 siRNAs, followed by transfection of MGRN1 or MGRN1ΔR. Cell lysates were immunoblotted to analyze the levels of endogenous LC3 II and p62 in the presence or absence of 300 nM bafilomycin A1. GAPDH was used as loading control. Efficiency of knockdown was confirmed by immunoblotting with anti-TSG101. Expression of MGRN1 or MGRN1ΔR was checked. Note that the expression of MGRN1ΔR phenocopies TSG101 depletion; also MGRN1 cannot rescue the effects mediated by TSG101. (**h**) Cells treated with mock or TSG101 siRNAs and transiently transfected with MGRN1 or MGRN1ΔR were subjected to Alexa-Fluor 488 EGF uptake. They were washed, fixed at indicated time points and imaged. Note that disruption of endo-lysosomal pathway by TSG101 depletion could not be salvaged by MGRN1 overexpression. Scale bar, 5 *μ*m. In the presence of TSG101 siRNA, overexpression of MGRN1ΔR generated a more severe qualitative phenotype than MGRN1

Some ESCRT proteins are known to facilitate vesicle fusion with lysosomes.^15, 16, 17, 18, 19, 20^ The ESCRT-I protein, TSG101 was the first identified ubiquitination substrate for MGRN1.^22^ It was obvious to ask if exogenous expression of TSG101 could rescue autophagic flux by restoring vesicular fusion (Figure 6a). Mock siRNA-treated cells, overexpressing control vector or HA-TSG101 showed comparable increase in the protein levels of LC3 II and p62 upon bafilomycin A1 treatment. Drug treatment did not significantly alter the autophagy proteins in MGRN1-depleted cells expressing control vector. HA-TSG101 overexpression, however, significantly enhanced LC3 II levels in bafilomycin A1-treated samples; p62 levels were also elevated (Figures 6b and c). It should be emphasized that though TSG101 is tightly regulated in cells,^40^ its ectopic expression reversed the MGRN1 depletion phenotype. Results were also corroborated in SHSY5Y cells using catalytically inactive MGRN1ΔR (Figures 6d and e). Exogenous HA-TSG101 expression rescued the dynamics of EGFR degradation (Figure 6f). In converse experiment, functional inactivation of TSG101 caused an increase in detectable LC3 II and p62; overexpression of MGRN1 or MGRN1ΔR did not affect this further, even if cells were treated with bafilomycin A1 (Figure 6g).

In parallel experiments to assay for EGF-induced EGFR uptake, trafficking and degradation, it was found that overexpression of MGRN1 could not rescue blocked clearance of vesicular cargo after TSG101 depletion (Figure 6h).

Hence, functional TSG101 was able to salvage the effects of MGRN1 inactivation, however, the reverse did not happen.

### Monoubiquitination of TSG101 affects vesicular fusion

**Figure 7:**
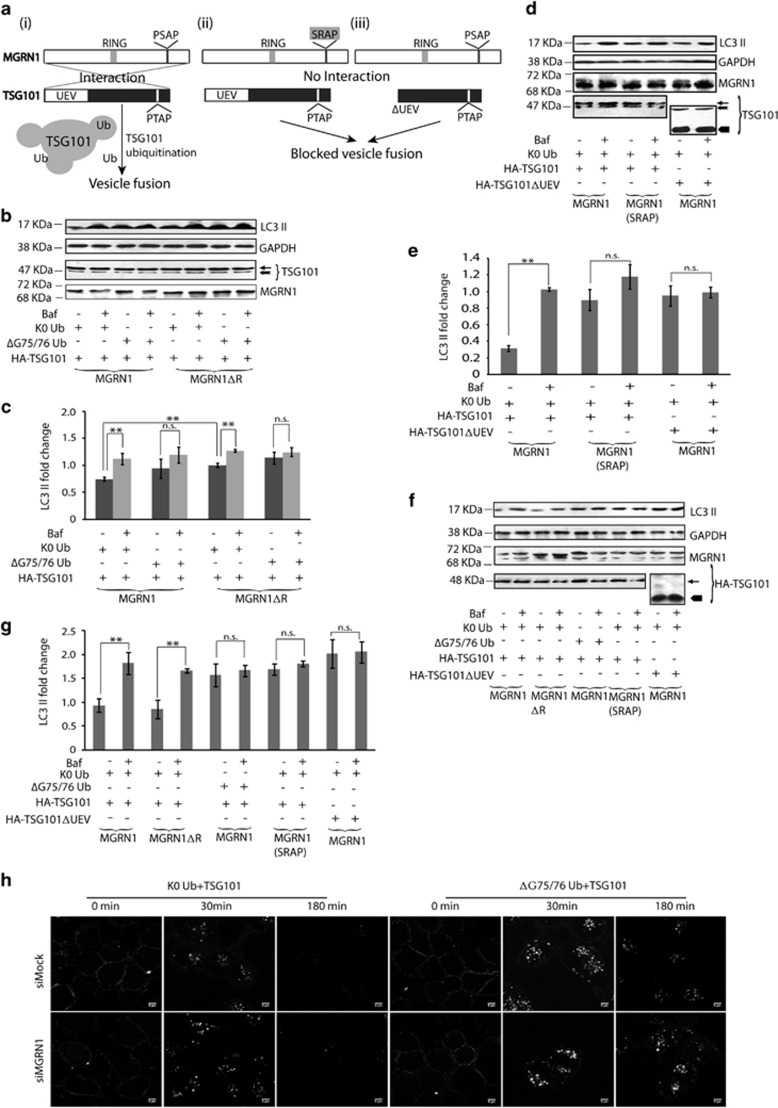
MGRN1-mediated monoubiquitination of TSG101 rescues lysosomal fusion events and restores degradation of cargo from autophagy and heterophagy pathways. (**a**) Experimental logic to establish that MGRN1 interacts with and ubiquitinates TSG101 to mediate vesicular fusion with lysosomes. (i) Monoubiquitination of TSG101 is essential for this. (ii) and (iii) Perturabation of the interacting regions of either MGRN1 (PSAP motif) or TSG101 (UEV domain) compromises vesicle fusions with lysosomes. (**b**) HeLa cells co-transfected with MGRN1 or MGRN1ΔR, HA-TSG101 along with K0 Ub or ΔG75/76 Ub were lysed and immunoblotted to analyze the levels of endogenous LC3 II in the presence or absence of 300 nM bafilomycin A1. Note that K0 Ub, a lysine-less ubiquitin mutant promotes only monoubiquitination; ΔG75/76 Ub cannot be conjugated to substrates, but binds noncovalently to ubiquitin interacting domains and acts as a competitive inhibitor of Ub binding. GAPDH was used as loading control. Efficiencies of all transfections were checked. The blots are representative of at least three experiments. Note that overexpression of TSG101, without its monoubiquitination cannot rescue autophagosomal degradation. 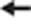 indicates HA-TSG101; 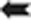 endogenous TSG101. (**c**) Quantification of data from (**b**) denotes fold change in endogenous LC3 II level when normalized against GAPDH from three independent experiments. **P≤0.05, n.s., not significant (*P*=0.3 and 0.6 in the presence of MGRN1 and MGRN1ΔR, respectively) using Student's *t*-test. Error bars, ±S.E.M. (**d**) HeLa cells co-transfected with MGRN1or MGRN1 (SRAP) along with HA-TSG101 or HA-TSG101ΔUEV and K0 Ub, as indicated, were lysed and immunoblotted to analyze the levels of endogenous LC3 II in the presence or absence of 300 nM bafilomycin A1. GAPDH was used as loading control. Efficiencies of all transfections were checked. Note that the lack of interaction between MGRN1 and TSG101 (when either MGRN1 (SRAP) or HA-TSG101ΔUEV was used) disrupts autophagosomal degradation. 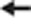 indicates HA-TSG101; 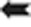 indicates endogeneous TSG101, 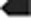 indicates HA-TSG101ΔUEV. (**e**) Graphical representation of LC3 II fold change as normalized against loading control. **P≤0.05, n.s., not significant (*P*=0.22 and 0.77 in the presence of MGRN1 (SRAP) and HA-TSG101ΔUEV, respectively) using Student's *t*-test. Error bars, ±S.E.M. (**f**) SHSY5Y cells co-transfected with MGRN1 or MGRN1ΔR, HA-TSG101 or HA-TSG101ΔUEV along with K0 Ub or ΔG75/76 Ub were lysed and immunoblotted to analyze the levels of endogenous LC3 II in the presence or absence of 60 nM bafilomycin A1. GAPDH was used as loading control. Efficiencies of all transfections were checked. The blots are representative of at least three experiments. Note that overexpression of TSG101, without its monoubiquitination cannot rescue autophagosomal degradation. 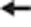 indicates HA-TSG101; 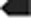 indicates HA-TSG101ΔUEV. (**g**) Graphical representation of LC3 II fold change as normalized against loading control. **P≤0.05, n.s., not significant (*P*=0.74, 0.45 and 0.92 in the presence of ΔG75/76 Ub,MGRN1 (SRAP) and HA-TSG101ΔUEV, respectively) using Student's *t*-test. Error bars, ±S.E.M. (**h**) HeLa cells treated with the indicated siRNAs, transiently co-transfected with HA-TSG101 along with K0 Ub or ΔG75/76 Ub was subjected to Alexa-Fluor 488 EGF uptake. Cells were washed, fixed at indicated time points and imaged. Note that overexpression of TSG101 and its monoubiquitination are required together to restore endo-lysosomal pathway. Scale bar, 5 *μ*m

MGRN1 is known to multi-monoubiquitinate TSG101, affect its solubility and hypothesized to have a key role in the pathogenesis of spongiform encephalopathy in *Mgrn1* null mice.^22, 41^ Induction of autophagic flux was used as a read-out to test the significance of MGRN1-mediated monoubiquitination of TSG101 in regulating vesicular fusion with lysosomes (Figure 7a(i)). In HeLa cells with bafilomycin A1 treatment, overexpression of TSG101 elicited significant increase in LC3 II levels in the presence of K0 Ub (a lysine-less ubiquitin, Ub mutant promoting only monoubiquitination)^42^ but not ΔG75/76 Ub (a competitive inhibitor of Ub)^43^ (Figures 7b and c). As in Figures 3 and 6, functional depletion of MGRN1 led to elevated levels of LC3 II.

It is known that MGRN1 interacts with TSG101 in a bimodal manner. One of these interactions involving the UEV (ubiquitin E2 variant) domain of TSG101 and the PSAP motif MGRN1 facilitates TSG101 monoubiquitination.^22^ To ascertain its significance in vesicular fusion, cells transfected with TSG101 or TSG101ΔUEV and K0 Ub along with MGRN1 or a PSAP motif mutant (MGRN1 (SRAP)) were assayed for autophagic flux (Figure 7a(ii and iii)). An increase in the levels of LC3 II with bafilomycin A1 treatment was observed only when K0 Ub, MGRN1 and TSG101 were present. Similar changes were not detected with either TSG101ΔUEV or MGRN1 (SRAP). These confirmed the importance of MGRN1-dependent monoubiquitination role of TSG101 in facilitating vesicular fusion in HeLa (Figures 7d and e) and SHSY5Y cells (Figures 7f and g).

Concurrently, heterophagy was also interrupted in MGRN1 siRNAs-treated HeLa cells transfected with TSG101 and ΔG75/76 Ub (Figure 7h).

## Discussion

**Figure 8:**
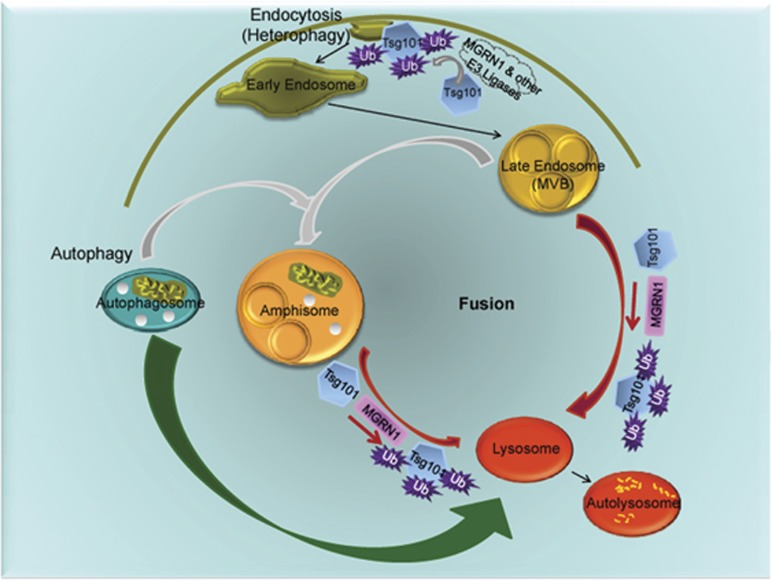
Schematic diagram summarizing the results. MGRN1-mediated multimonoubiquitination of TSG101 governs fusion between lysosomes and amphisosomes/late endosomes (denoted by red arrows); MGRN1 does not affect fusion between autophagosome and late endosome. Role of MGRN1 in direct fusion between autophagosome and lysosomes remains a possibility. Grey arrows denote pathways where role of MGRN1 is not implicated. Green arrow denotes pathway where role of MGRN1 is unknown

This study elucidates the mechanism by which MGRN1 regulates lysosomal degradation by simultaneously affecting autophagy and heterophagy. In cell lines and primary cells, functional inactivation of MGRN1 led to an increase in the number and size of LC3 positive vesicles, indicating altered autophagy. In cells pushed towards neuronal differentiation, depletion of MGRN1 resulted in blocked fusion between autophagosomes (via amphisomes) and lysosomes. Compromised fusion was detected in the presence of ^Ctm^PrP or cyPrP, constructs that are suggested to decrease MGRN1 activity. The autophagic flux and degradation competence were affected. Also disrupted were endo-lysosomal trafficking and degradation of EGFR, resulting in an accumulation of amphisomes. This highlights a role for MGRN1 in the fusion of amphisome/MVBs with lysosomes – thus modulating both autophagosomal–lysosomal and endo-lysosomal degradations. MGRN1 monoubiquitinates TSG101 to facilitate these fusion events. (Figure 8). Whether MGRN1 regulates autophagosomal–lysosomal fusion needs to be addressed.

The ESCRT proteins are important for recognition of ubiquitinated integral membrane proteins to be endocytosed, maturation into MVBs and subsequent degradation at the lysosomes. The significance of these proteins in heterophagic degradation has been suggested in yeast and mammalian systems.^44, 45, 46^ However, our understanding of the role of these proteins in autophagic degradation is still premature.

Mutations in the ESCRT-III subunit, CHMP2B (charged multivesicular body protein 2B or chromatin-modifying protein 2B)/Vps2B are reported in patients with neurodegenerative diseases, like FTD^47^ and ALS.^48^ Similar mutations result in accumulation of aggregates containing ubiquitinated proteins, p62 and Alfy (autophagy-linked FYVE protein)^15^ – key components of autophagy. Efficient clearance of such aggregates requires functional MVBs. This suggests that CHMP2B, along with other ESCRT subunits, has a significant role in abating accumulation of abnormal proteins, disruption of neuronal activity and ultimately neurodegeneration by regulating fusion of the autophagosomes with endosomes and lysosomes.

Intuitively, inhibition of such fusion events should cause accumulation of autophagosomes and corresponding decrease in amphisomes and autolysosomes as detected in varied experimental systems.^16, 17, 49, 50, 51^ However, there is compelling evidence to suggest that abrogation of normal ESCRT activity can result in increased numbers of amphisomes and autolysosomes.^15, 18^

Homozygous knockout animal for the ESCRT-I protein, TSG101, is embryonic lethal.^52^ Conditional TSG101 deletions in cellular systems show enlarged lysosomes enriched with LC3 without perturbation of LAMP-1 or CTSD trafficking and localization,^53^ suggesting its involvement autophagy. However, the mechanism for the enrichment of autophagy proteins in LAMP-1 positive vesicles is yet unclear. This could be due to the enrichment of amphisomes positive for LC3 and LAMP-1.

On similar lines, our present study identifies that catalytic inactivation of MGRN1 affects TSG101 to disrupt amphisome–lysosome and MVB–lysosome fusion. The formation of amphisomes (generated by fusion between autophagosomes and MVBs) remains unperturbed. This would imply that each of the fusion events is distinct, involving separate molecular players.

It is known that TSG101 may get ubiquitinated by other E3 ligases – MDM2 and TAL.^54, 55^ It cannot be completely ruled out that it probably gets ubiquitinated by one of these ligases when MGRN1 is non-functional. In the brains of young *Mgrn1* null mutant mice ubiquitinated TSG101 is reduced but not absent.^41^ Hence, ubiquitination of TSG101 is a defining event in regulating cargo clearance at the lysosomes. It is perfectly justified to extrapolate that simultaneous modulation of the two very heavily utilized arms of lysosomal degradation by the ubiquitously expressed MGRN1 could govern neurodegeneration in some types of prion diseases.

## Materials and Methods

### Constructs, antibodies and reagents

MGRN1, MGRN1ΔR, WT PrP, PrP(A117V), PrP(KH II), PrP(AV3), SA PrP, Ifn PrP constructs have been described before.^23^ mCherry-EGFP-LC3B was gift of Terje Johansen; HA-Ub and HA-ΔG75/76 ubiquitin were gifts of Rafael Mattera; HA-K0 ubiquitin was gift of Kah-Leong Lim; Ub^G76V^-GFP was a gift of Nico P Dantuma, HA-tagged TSG101 and GFP-tagged TSG101 were gifts of Juan S Bonifacino; GFP-LC3 was gift of Nitai P Bhattacharyya. RFP-LC3, HA-TSG101ΔUEV and MGRN1 (SRAP) were generated using standard cloning and mutation techniques.

Antibodies were from the following sources: CD63 (BD Pharmingen, #556019, San Jose, CA, USA), LAMP2 (H4B4, Hybridoma Technology, Iowa City, IA, USA), Beclin1 (Novus Biologicals, #NB500-249, Littleton, CO, USA), LC3 (Novus Biologicals, #NB100-2220), p62/ SQSTM1 (Thermo Scientific, #PA5-20839, Rockford, IL, USA), *β-*catenin (Cell Signaling Technology, #9562, Danvers, MA, USA), Monoclonal anti GAPDH clone GAPDH-71.1 (Sigma-Aldrich, #G8795, St. Louis, MO, USA), EGFR (A10) (Santa Cruz Biotechnology, Inc., #sc-373746, Dallas, TX, USA), Rab7 (Abcam, #ab50533, Cambridge, UK), *β*-tubulin (Abcam, #ab7792), TSG101(Abcam, #ab83), Ub (Sigma-Aldrich, #U0508, Anti-Cathepsin D (CTD-19, Abcam, #ab6313)). The MGRN1, PrP, GFP and HA antibodies were gifts of Ramanujan S Hegde (Cambridge, UK).

MG132 (Sigma-Aldrich, #C2211) was used at 10 *μ*M concentration for 6 h. Bafilomycin A1 (Sigma-Aldrich, #B1793) was used at 300 nM concentration for 10 h for HeLa, 60 nM concentration for 7 h for SHSY5Y and 50 nM concentration for 4 h for Melan cells. Rapamycin (Sigma-Aldrich, #R0395) was used at 200 nM concentration for HeLa for 24 h. All-*trans*-retinoic acid (Sigma-Aldrich, #R2625) was used at 10 *μ*M concentration for 4 days. Recombinant HuEGF (Gibco, #PHG0311, Frederick, MD, USA) at 100 ng/ml and Alexa-Fluor 488 EGF (Invitrogen, #E-13345, Eugene, OR, USA) at 3 *μ*g/ml concentrations were used. Pepstatin A (Sigma-Aldrich, #P5318) at 10 *μ*g/ml concentration; Monensin (Sigma-Aldrich, #M5273) at 10 *μ*M; Nigericin (Sigma-Aldrich, #N7143) at 20 *μ*M concentration were used.

### Cell culture and immunocytochemistry

Cell lines used for the experiments were HeLa (human cervical cancer cell line), SHSY5Y (human neuroblastoma), MEFs (mouse embryonic fibroblast cells), immortal melanocytes (control melan-a6 or *Mgrn1* null mutations, melan md1-nc). HeLa, SHSY5Y and MEF cells were grown in 10% fetal bovine serum (FBS; Gibco, Grand Island, NY, USA)/Dulbecco's modified Eagle's medium (DMEM; Himedia, Mumbai, India) media at 37 °C and 5% CO_2_. Immortal melanocytes were grown in 10% fetal calf serum (FCS; Gibco, #10270-106)/RPMI-1640 (Gibco, #31800-022)/200 nM 12-*O*-tetradecanoylphorbol 13-acetate (TPA; Sigma-Aldrich, #P8139) media at 37 °C and 10% CO_2_, as per the guidelines of the Wellcome Trust Functional Genomics Cell Bank. MEF (CF-1 strain) was gift of Mitradas M Panicker, Bengaluru, India. Immortal melanocytes (gift of Ramanujan S Hegde) were obtained from the Wellcome Trust Functional Genomics Cell Bank.

SHSY5Y cells were treated with 10 *μ*M all-*trans*-retinoic acid for 4 days in DMEM and 10% heat inactivated FBS to induce them to differentiate into cells of neuronal fate.^32^

Cells were transfected with DNA at 90% confluency using Lipofectamine 2000 (Invitrogen, Carlsbad, CA, USA) as per the manufacturer's instructions. For transient transfection with various constructs, 24 h post-transfected cells were undergone treatment if required for specified time points and then samples were prepared for imaging or biochemical analysis.

All tissue culture plastic wares and Lab-Tek 8-well chambered slides used for microscopy were from Nunc, Rosklide, Denmark, and bottom coverglass dishes used for microscopy were from SPL Lifesciences, Gyeonggi-do, Korea.

Immunohistochemistry was done with minor modifications of earlier methods.^23^ In brief, cells were fixed with either 10% formaldehyde or methanol as per the requirement of the Ab. Cells were permeabilized using 10% FBS/phosphate buffer saline (PBS)/0.1% saponin (Sigma-Aldrich, #S4521) for 60 min, followed by overnight staining in primary Ab at 4 °C and 60 min of incubation in secondary Ab at room temperature. The samples were then imaged using confocal microscope. For tagged construct, cells were either fixed or seen directly (in case of live cell imaging) under microscope. Cells were incubated in CO_2_-independent medium (Gibco, #18045-088) while performing live cell microscopy.

### Knockdowns with siRNA

ON-TARGETplus SMARTpool siRNAs against MGRN1 (L-022620-00-0020), human TSG101 (L-003549-00-0005), non-targeting siRNA (D-001810-01-20), GFP (D-001300-01-20) acquired from Thermo Scientific Dharmacon Products, Lafayette, CO, USA) were transfected using Lipofectamine 2000 following the manufacturer's instructions. In brief, 50% confluent cells were treated with siRNA for a total of 72 h and then samples were prepared either for imaging or for biochemical assay. Cells to be imaged were divided into two parts, trypsinized and replated after 48 h – one set fixed another 24 h after this, permeabilized and stained for immunocytochemistry, while the second set lysed to check for knockdown efficiency biochemically.

For experiments where cells needed transfection after siRNA treatments, they were first transfected with siRNA for 48 h followed by trypsinisation and replating in smaller dishes, Cells were then retransfected with various contructs for 20 h and treatments were done as indicated on the following day. Cells were then harvested for biochemical analyses or used for imaging (as live or fixed samples, as per experimental requirements). This allows similar knockdown throughout the sample set for individual experiment.

### Western blotting

The protocol for western blotting was as before.^23^ In brief, cells were washed with PBS and lysed on ice in lysis buffer (1M Tris-HCl, pH 7.5, 1 N NaCl, 0.5 M EDTA, 1M NaF, 1M Na_3_VO_4_, 10% SDS, 200 mM PMSF, 10% Triton X-100, 50% glycerol) for 30 min in presence of complete protease inhibitor (Sigma-Aldrich, #P8340) and centrifuged at 13 000 r.p.m. for 15 min. Protein concentration was determined by Bradford protein estimation assay. Tris-tricine gels (7.5, 10 or 12%) were run as per the molecular weight of the proteins being probed. Transfer and immunoblotting were done as per the standard procedures. Quantification of western blots was carried out using Quantity One software of Bio-Rad. At least three independent experiments were analyzed and band intensities were normalized to loading control. *P*-values were determined using Student's *t*-test.

The interpretation of autophagic flux by checking the levels of LC3 II and p62, in the presence or absence of inhibitors was as analyzed previously.^56^

### Lysotracker assay

Melan cells were washed with PBS and stained with 500 nM LysoTracker Red DND-99 (Molecular Probes, #L-7528, Eugene, OR, USA) in DMEM for 30 min at 37 °C, rinsed with cold 1 × PBS (4 °C), and fixed with 10% formaldehyde in PBS for 10 min at room temperature before imaging.

### *In vivo* ubiquitination assay

*In vivo* ubiquitination assays were performed as described previously.^22^ In brief, HeLa cells were transiently co-transfected with GFP-tagged TSG101 and HA-tagged ubiquitin (Ub) along with MGRN1, MGRN1ΔR, WT PrP or PrP(A117V). 24h post transfection cell lysates were immunoprecipitated with anti-GFP antibody. Ubiquitinated TSG101 was detected by immunoblotting with anti-Ub antibody. Cell lystaes were also analyzed for the expressions of GFP-TSG101, MGRN1 and PrP.

### EGFR endocytic trafficking assay

For measurement of EGF endocytic trafficking, cells treated with siRNAs and transfected with various constructs as (described before) were subjected to serum withdrawal for 4 h followed by cold PBS wash. Cells were then incubated with 3 *μ*g/ml Alexa-Fluor 488 EGF in DMEM/1%BSA (Sigma-Aldrich, #A7906)/20 nM HEPES for 1 h on ice. Cells were then washed and further incubated with pre-warmed DMEM/10% FBS for indicated time periods. After incubation, cells were washed with ice-cold PBS and immediately fixed with 10% formaldehyde and imaged.

### EGFR degradation assay

HeLa cells treated with siRNAs and transfected with various constructs as (described before) were starved for 5 h and incubated with 100 ng/ml EGF in DMEM/ 1% BSA for 0, 30 min and 3 h. Cells were then washed with cold PBS and lysed in lysis buffer.

### Lysosomal activity assay

Lysosomal activity was analyzed using Cathepsin D Activity Assay Kit (Fluorometric; Abcam, #ab65302) as per the manufacturer's instruction. In brief, cells were lysed in the buffer provided in the kit and mixed with appropriate volumes of reaction buffer and substrate, followed by 2 h incubation at 37 °C. Samples were prepared from HeLa cells either treated with control or MGRN1 siRNAs. To block CTSD activity, 56 h after adding siRNAs, cells were treated with either vehicle control or 10 *μ*g/ml pepstatin A for 16 h before sample preparation, as indicated. On the day of experiment, cells were counted using a heamocytometer and lysates prepared from three different dilutions of indicated cell concentrations. Number of technical replicates for each cell concentration is three and the experiment was replicated thrice. Data acquisition was done using Varioskan Flash Multimode Reader (Thermo Scientific).

### Fluorescence microscopy and imaging

Fluorescence microscopy was performed utilizing LSM510-Meta and LSM710/ConfoCor 3 microscopy systems (Zeiss, Jena, Germany) and Nikon microscope equipped with an Ar–ion laser (for GFP excitationor Alexa-Fluor 488 with the 488 nm line) a helium–neon (He–Ne) laser (Alexa-Fluor 546 and 594 excitation with the 543 line). For all imaging, 63*1.4 numerical aperture (NA) oil immersion objective was used. For time course experiments, live cells were imaged as per established protocol.^57^ For quantitative analyses and comparisons between multiple samples, images were collected using identical excitation and detection settings.

The detector gain settings were chosen to allow imaging of the desired cells within the linear range of the photomultiplier tube without saturating pixels, unless otherwise specified. Using ImageJ, the RFP-LC3 and GFP-LC3 and lysotracker images were converted to black and white images using the threshold function, and the vesicles diameter for each lysosome was manually measured as previously described.^23^ For each lysosome, diameters were measured along two random axes to minimize the scope of manual errors likely to skew results.

Confocal imaging of MGRN knockdown samples in Figures 1a–c and S1 were done using the following parameters: digital gain for green and red channel, respectively, 750 and 800, laser power for green and red channel, respectively, 5 and 10%, digital offset:1.

In experiments where mCherry-EGFP-LC3B construct was used, vesicles were counted to calculate the percentage of red vesicles among the total number of vesicles (yellow±red); the average percentage (percentage divided by the total number of cell=*n*) was used to plot the graph. For this, the following equation was used:

Percentage of red vesicles=(average number of red vesicles in *n* cells/average number of (yellow±red) vesicles) × 100

Confocal imaging of CD63 immunostained samples in Figure 5a was done using the Nikon A1R± Ti-E with N-SIM and FCS microscope systems.

The data were tabulated in Microsoft Excel, which was used to generate the histograms and perform statistical analyses by the Student's *t*-test.

### Analysis of vesicles

Random fields of immunostained cells with various vesicular markers/antibodies were chosen and imaged. 15–20 fields for each condition, containing at least four cells, were imaged. Using ImageJ, the images were converted to black and white images using the threshold function, and the vesicular diameter for each was manually measured, in a protocol similar to that used for quantitation of lysosomal sizes.^23^ For each vesicle, diameters were measured along two random axes to minimize the scope of manual errors likely to skew results. The data were tabulated in Microsoft Excel, which was used to generate the histograms and perform statistical analyses by the Student's two-tailed *t*-test.

### Lysosomal pH measurement

LysoSensor Yellow/Blue dextran (Molecular Probes, #L-7545) was used for measuring cellular pH using both flourometric and confocal microscopy approach as described before.^58, 59^ This dextran-linked dye is known to accumulate in endocytic vesicles (endosomes and lysosomes) through endoytosis and exihibits pH-dependent dual-emission spectra – with an emission maximum of 530 nm at acidic pH, while at higher pH the emission is maximum at 450 nm.

In brief, cells were grown in 96-well plate and 50% confluent cells were treated with 0.5 mg/ml of the pH indicator LysoSensor Yellow/Blue dextran for 16 h. The standard curve was generated by incubating cells in 10 *μ*M monensin and 20 *μ*M nigericin for 10 min in MES buffer (5 mM NaCl, 115 mM KCl, 1.2 mM MgSO_4_, 25 mM 4-morpholineethanesulfonic acid), with the pH adjusted to a range from 3.5 to 7.0. The samples were then read in a Varioskan Flash Multimode Reader (Thermo Scientific) with excitation at 360 nm. The ratio of emission 530/450 nm was then calculated for each sample. siRNA-treated cells, 56 h post transfection were incubated with LysoSensor Yellow/Blue dextran for additional 16 h, washed with cold PBS and read in the plate reader. pH values for control and MGRN1 siRNA-treated samples were determined from the standard curve generated via the pH calibration samples.

For pH measurement using confocal microscopy, all conditions were similar except the indicator dye was used at 1 mg/ml concentration.^59^ LysoSensor Yellow/Blue dextran-treated cells were imaged using a single excitation filter of 360 nm and two emission filters at 450 and 515 nm. Dye emission at 530 nm was assigned the color red (R), whereas the emission at 450 nm was assigned the color green (G). The ratio of 530 to 450 provided a measurement of pH. With a standard pH calibration curve as reference, the pH of endocytic vesicles was calculated using Image J; ~200 vesicles were counted to first establish a pH titration curve. siRNA-treated cells, 56 h post transfection were incubated with the pH indicator LysoSensor Yellow/Blue dextran for 16 h, washed with cold PBS, fixed with 10% formaldehyde, imaged and analyzed using identical parameters as used for establishing the titration curve. In representative images, the red and green colors were converted to magenta and blue, respectively, using LUT in ImageJ.

## Abbreviations

Ab: antibody
A*β*: amyloid *β-*peptide
Alfy: autophagy-linked FYVE protein
ALS: amyotropic lateral sclerosis
APP: amyloid precusor protein
ATG: autophagy-related gene
Baf: bafilomycin A1
BECN1: Beclin1
CHMP2B: charged multivesicular body protein 2B
CTSD: Cathepsin D
^Ctm^PrP: C-terminal transmembrane form of prion protein
cyPrP: cytosolic prion
DMEM: Dulbecco's Modified Eagle Medium
EGF: epithelial growth factor
EGFR: epithelial growth factor receptor
EGFP: enhanced green fluorescent protein
ESCRT: endosomal sorting complex required for transport
ErbB2: v-erb-b2 avian erythroblastic leukemia viral oncogene homolog 2
FBS: fetal bovine serum
FCS: fetal calf serum
FTD: frontotemporal dementia
GFP: green fluorescent protein
HA: hemagglutinin
He-Ne: helium–neon
HTT: Huntingtin
Hu: human
ILV: intralumenal vesicles
LC3: microtubule-associated protein 1 light chain 3
LUT: lookup table
MEFs: mouse embryonic fibroblasts
MES: 4-morpholineethanesulfonic acid
MGRN1: Mahogunin Ring Finger-1
MGRN1ΔR: Mahogunin Ring Finger-1 delta RING mutant
MVB: multivesicular body
NA: numerical aperture
Opn: osteopontin
p62: protein62
PBS: phosphate buffer saline
PMSF: 14 piperazinediethanesulphonic acid
PrP: prion protein
PrP^Sc^: scrapie form of prion protein
RA: all-*trans*-retinoic acid
RFP: red fluorescent protein
RFU: relative fluorescence units
RING: really interesting new gene
r.p.m.: revolutions per minute
RPMI: Roswell Park Memorial Institute medium
SDS-PAGE: sodium dodecyle sulphate-polyacrylamide gel electrophoresis
siRNA: small interfering ribonucleic acid
TPA: 12-*O*-tetradecanoylphorbol 13-acetate
TSG101: tumor suppressor gene101
Ub: ubiquitin
UEV: ubiquitin E2 variant
UPS: ubiquitin-proteosome system
