# Supplementary figures and images for: Mahogunin regulates fusion between amphisomes/MVBs and lysosomes via ubiquitination of TSG101
Source: Cell Death Dis. 2015 Nov 5;6(11):e1970–. doi: 10.1038/cddis.2015.257 (PMC4670916; doi:10.1038/cddis.2015.257)

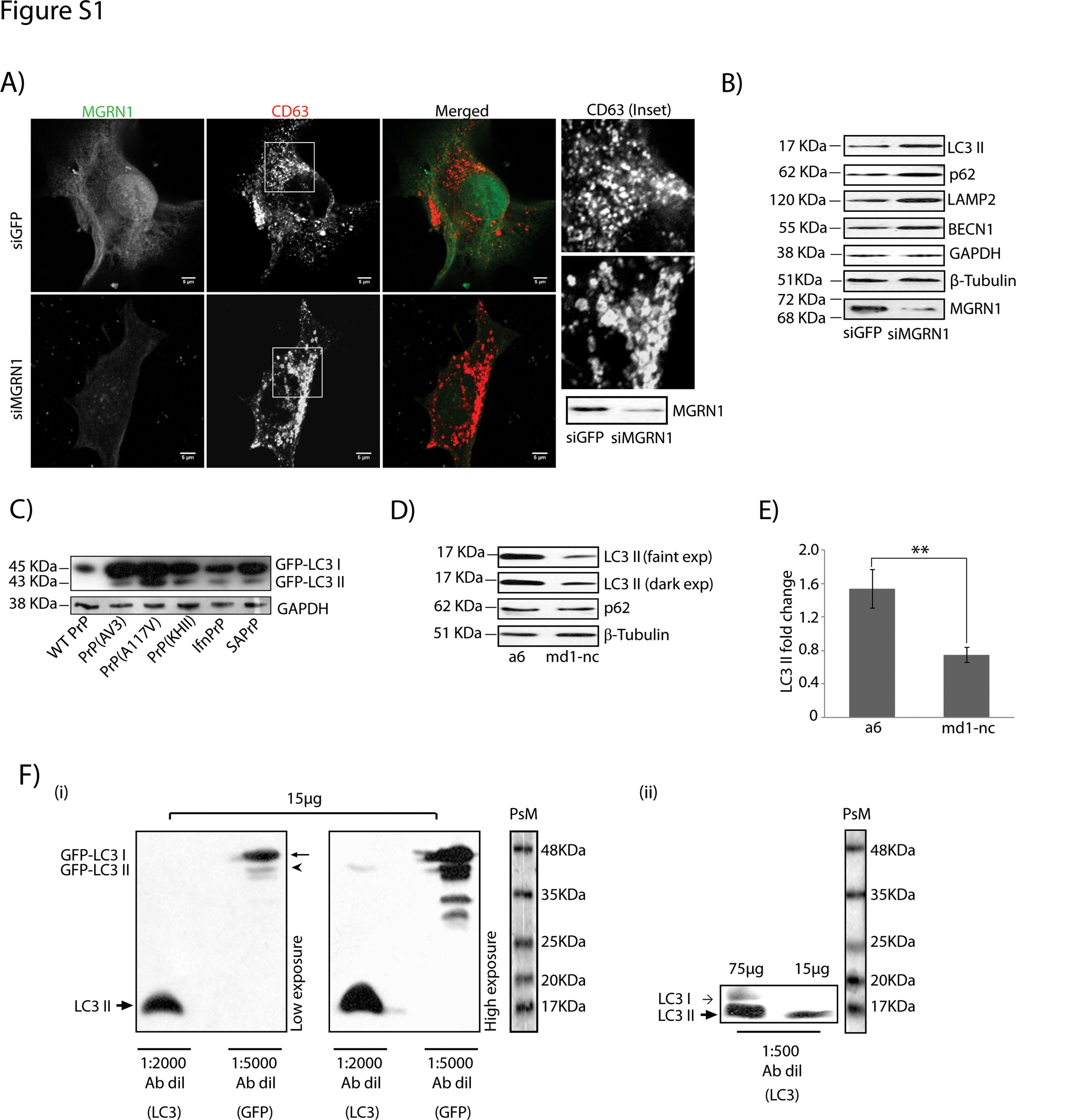

Supplement: Supplementary Figure 1 [file cddis2015257x1.tif]

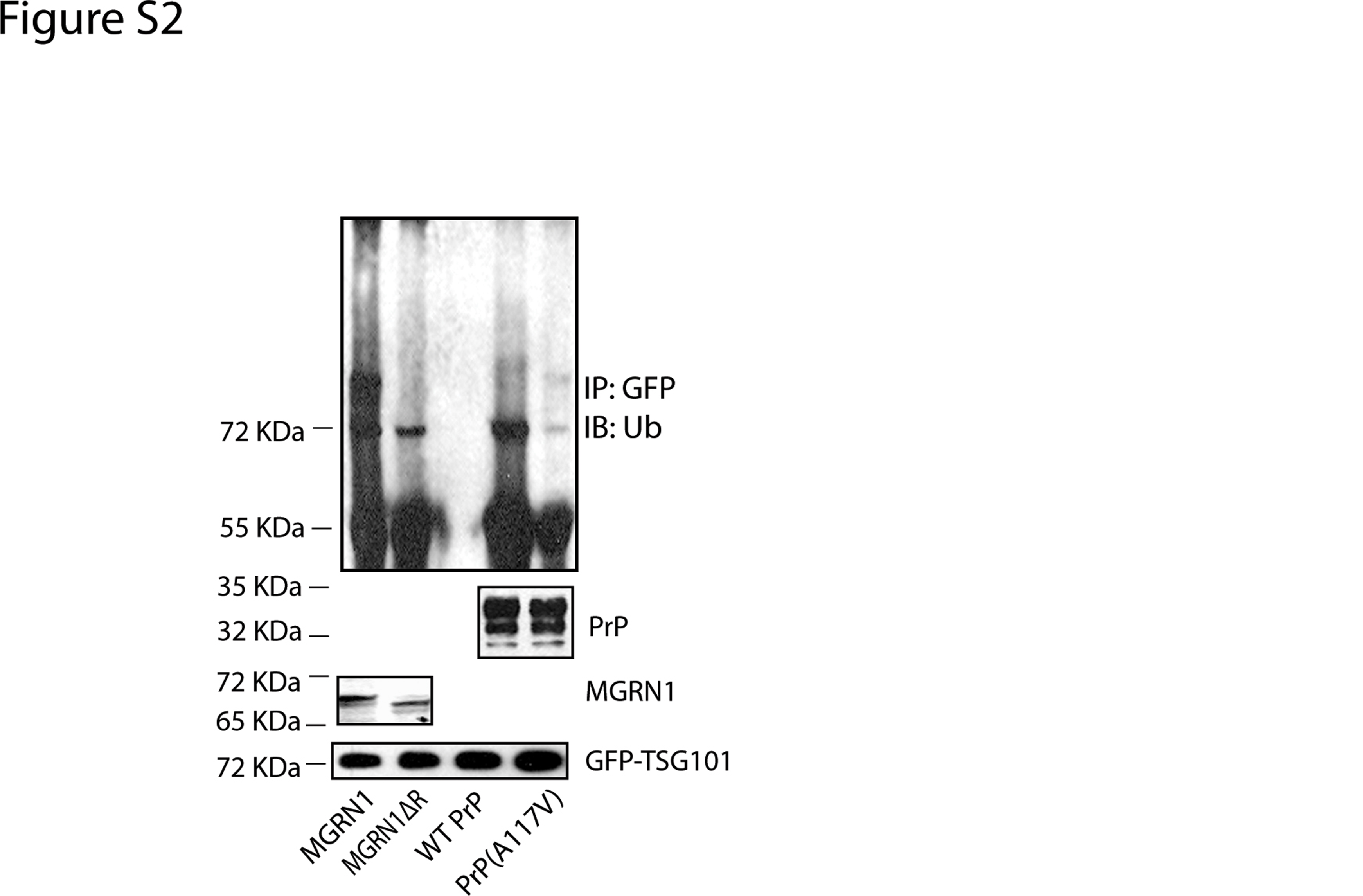

Supplement: Supplementary Figure 2 [file cddis2015257x2.tif]

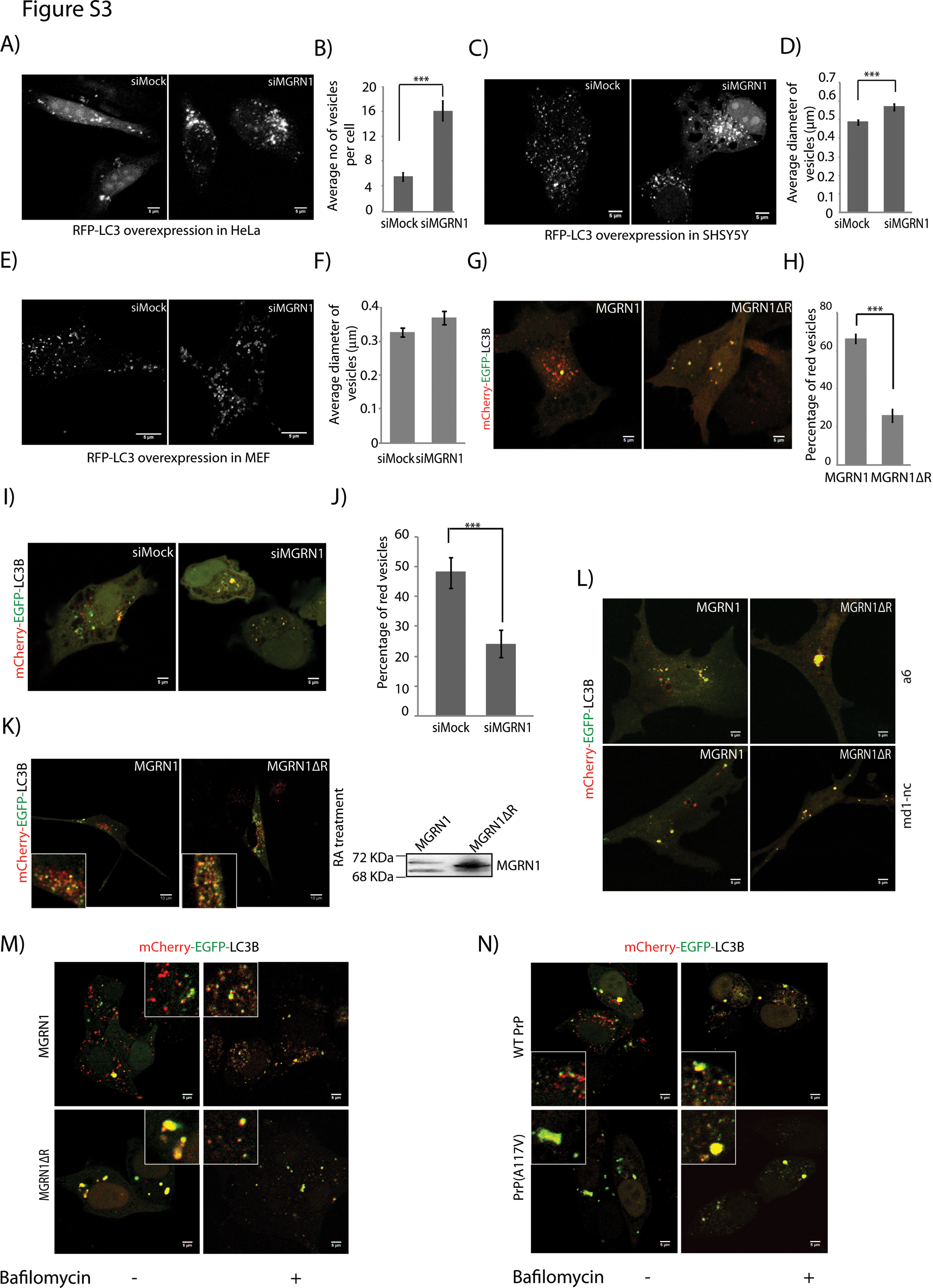

Supplement: Supplementary Figure 3 [file cddis2015257x3.tif]

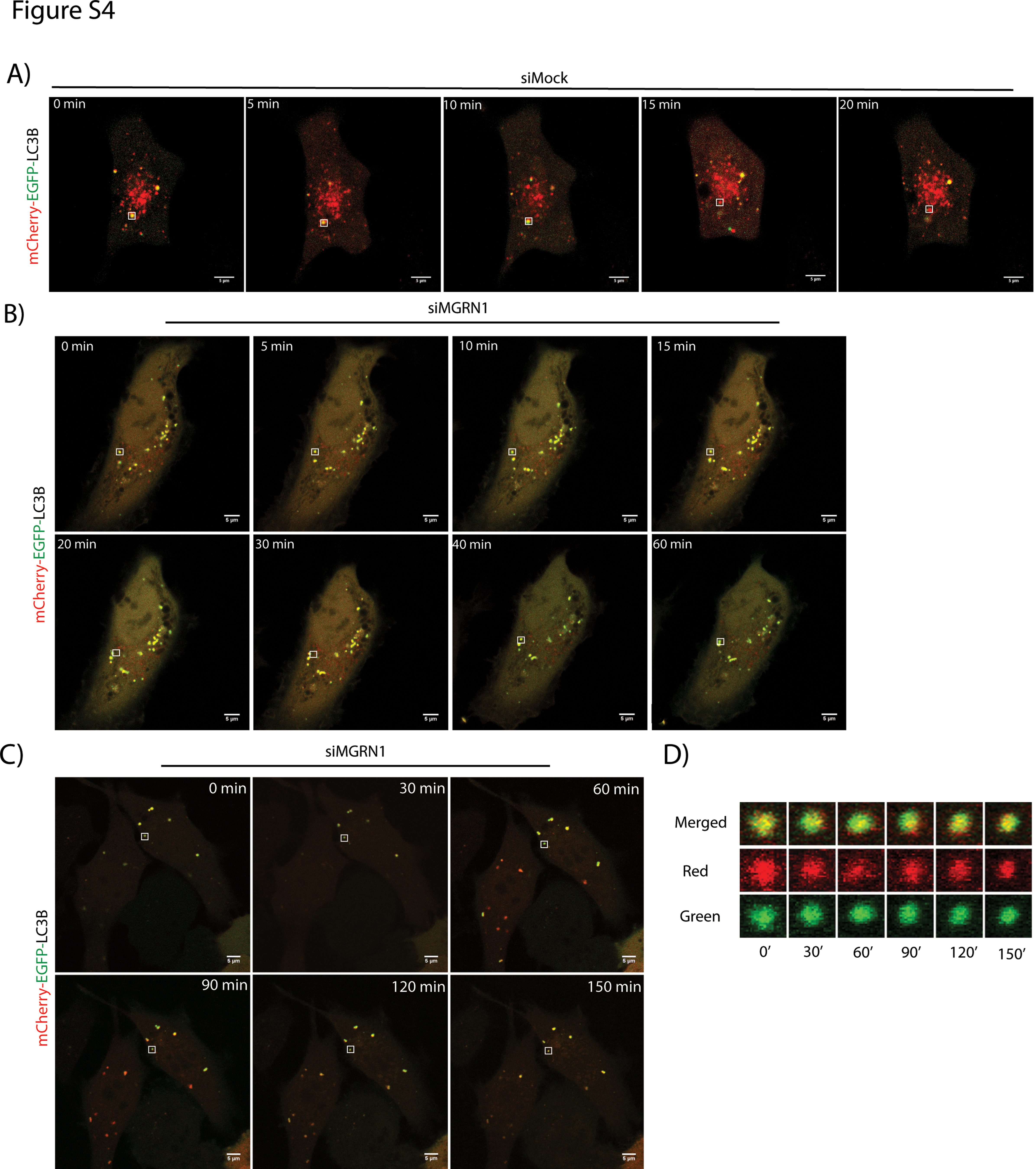

Supplement: Supplementary Figure 4 [file cddis2015257x4.tif]

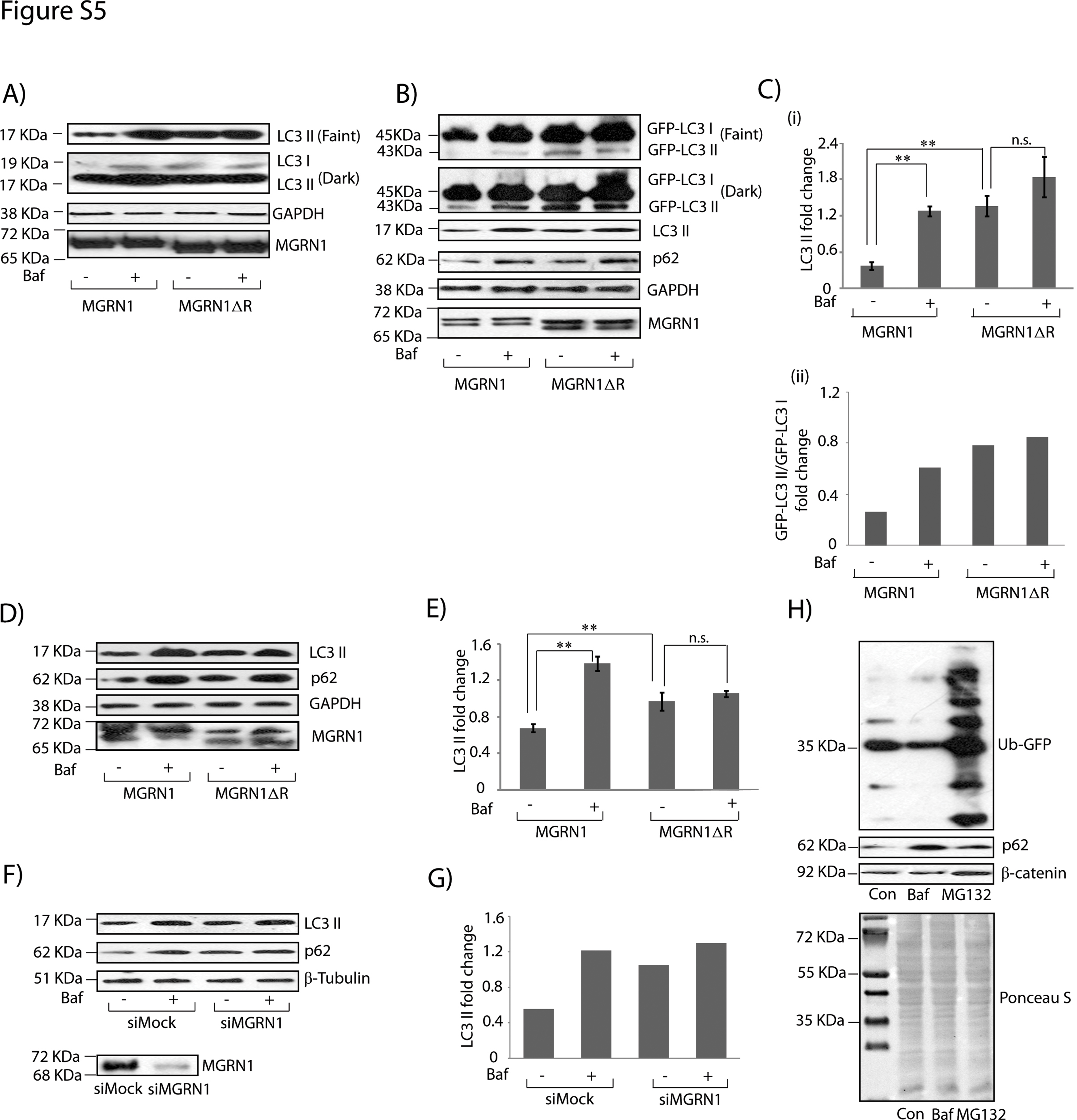

Supplement: Supplementary Figure 5 [file cddis2015257x5.tif]

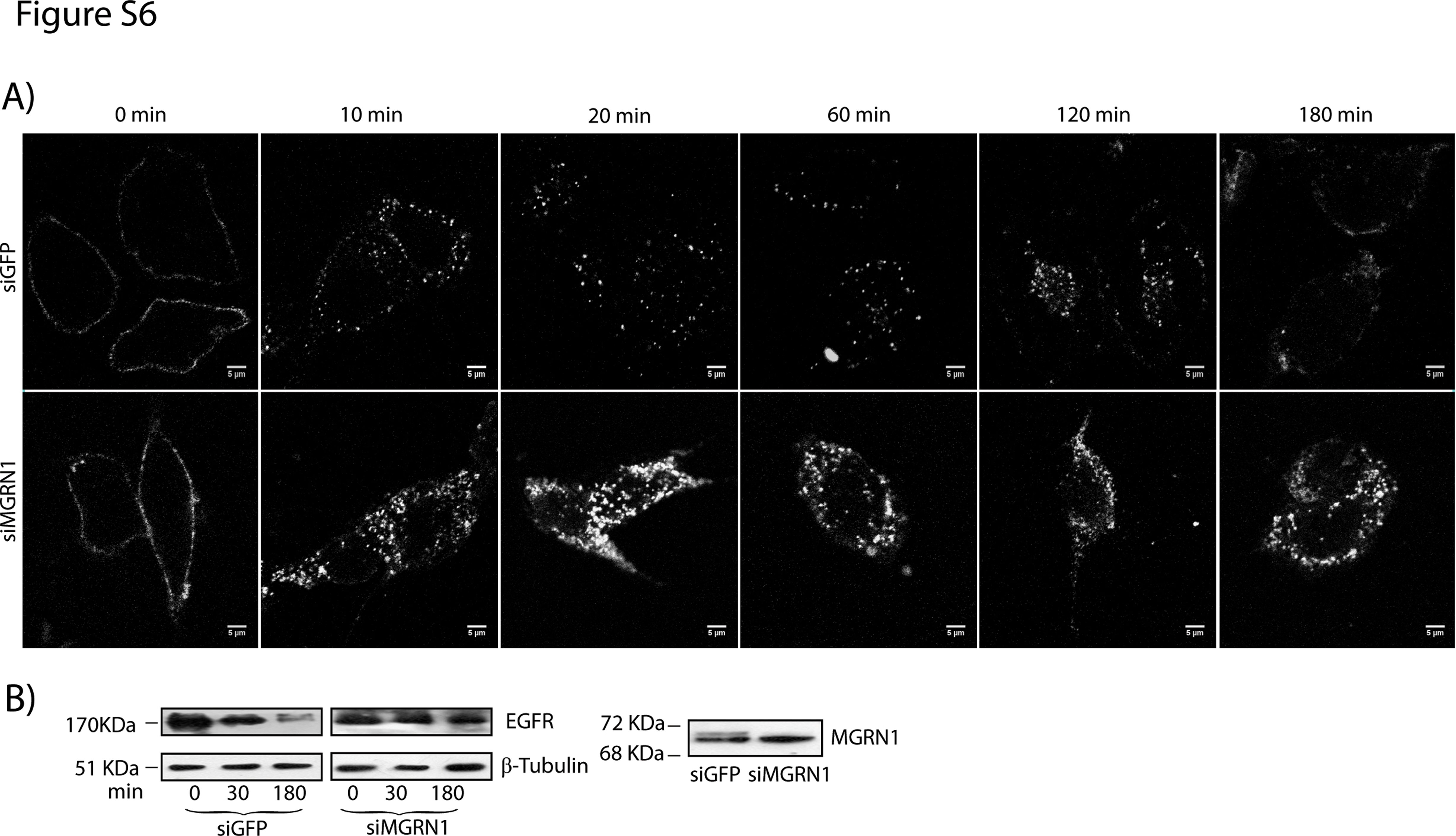

Supplement: Supplementary Figure 6 [file cddis2015257x6.tif]

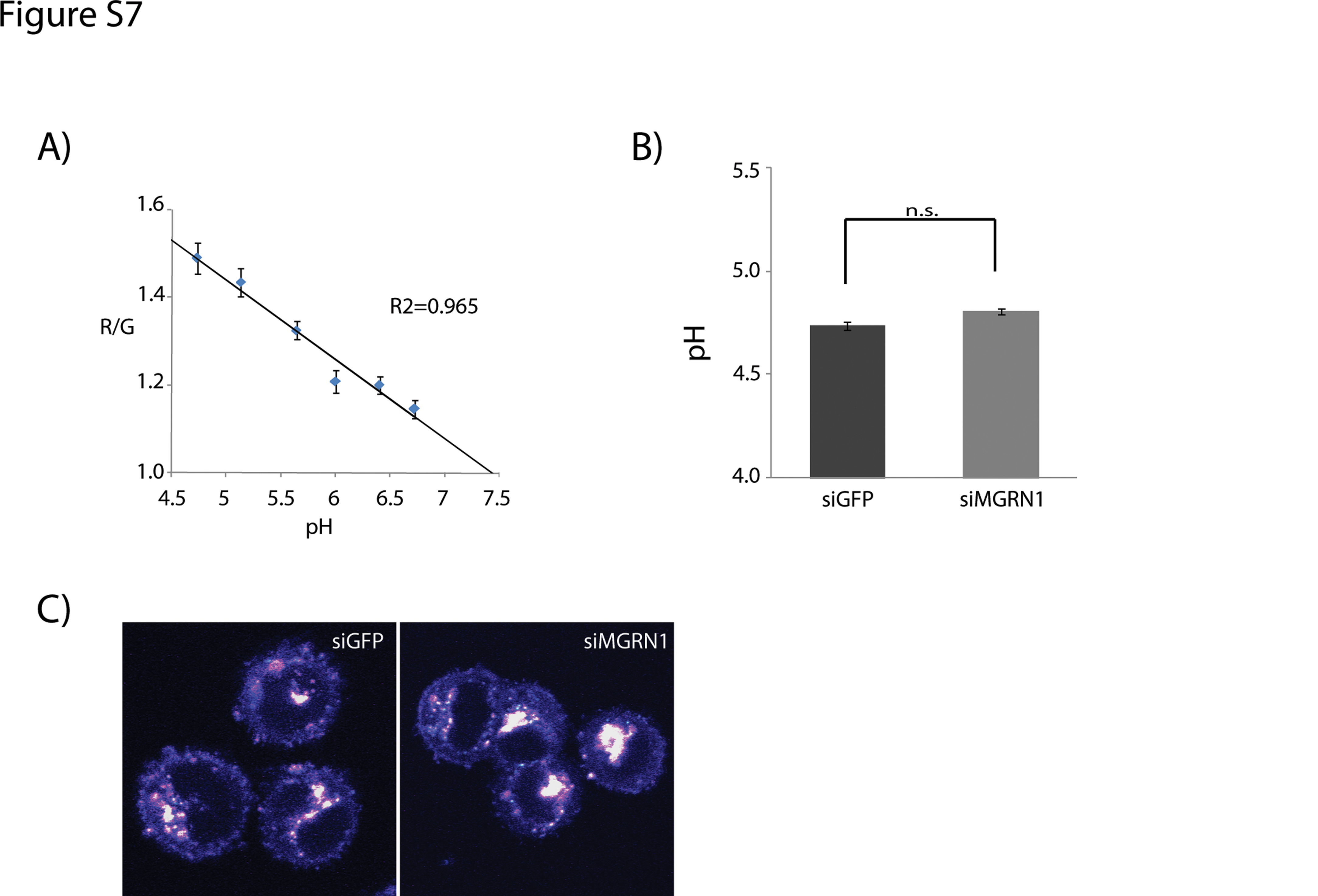

Supplement: Supplementary Figure 7 [file cddis2015257x7.tif]
