# Supplementary material for: Mahogunin regulates fusion between amphisomes/MVBs and lysosomes via ubiquitination of TSG101
Source: Cell Death Dis. 2015 Nov 5;6(11):e1970–. doi: 10.1038/cddis.2015.257 (PMC4670916; doi:10.1038/cddis.2015.257)
Supplement: Supplementary Information [file cddis2015257x8.doc]

**Supplementary text**

**Figure S1: Expression of markers of late endosome/lysosome in cell lines.**

(A) SHSY5Y cells treated with irrelevant siRNAs (GFP siRNAs) or MGRN1 siRNAs, co-immunostained for CD63 and MGRN1 were imaged in a similar experiment as in Figure 1A. Insets show enlarged view of the areas within white boxes. The immunoblots on the right show efficient siRNA mediated MGRN1 knockdown. Note a qualitative increase in size of vesicles with the functional depletion of MGRN1. The channels for acquiring the images are indicated. Scale bar, 5μm.

(B) SHSY5Y cells similarly treated with siRNAs were lyzed and immunoblotted for autophagy and lysosomal proteins. Efficiency of knockdown was shown using anti-MGRN1 antibody.

(C) HeLa cells transiently co-transfected with GFP-LC3 and various indicated PrP mutants were lysed and immunoblotted to check the levels of GFP-LC3 I and II in them.

(D) melan a-6 and melan md1-nc cell lysates were immunoblotted for autophagy marker proteins. Faint and dark exposures for the LC3 II blot are shown for ease of understanding. All blots are representative of at least 3 independent experiments. The levels of GAPDH or β–tubulin serve as loading control.

(E) Histogram depicts fold change in LC3 II in melan md1-nc cells as compared with the control melan a-6 cells, when normalized against corresponding β–tubulin. Graph shows results from 3 independent experiments. ** p ≤ 0.05, using Student’s t-test. Error bars, +SEM.

(F) Detection of LC3 I and II forms. (i) The antibody used efficiently detects LC3 II levels from cells lysates. HeLa cells transiently transfected with GFP-LC3 were lysed to check for the levels of endogenous LC3 and exogenously expressed GFP-LC3. 15 µg of lysate was loaded on a 12% Tris-Tricine SDS-PAGE in duplicate on either side of a lane containing pre-stained marker (PsM). The blot was cut through the middle of the marker band and the two halves were immunoblotted with LC3 and GFP antibodies. Using 1:2000 dilution of the LC3 antibody, only the endogenous LC3 II form could be detected on low exposure. With a high exposure, however, GFP-LC3 II could also be detected -- thus indicating a higher affinity of this antibody for the LC3 II forms. GFP antibody could detect both LC3 I and II forms even on low exposure. (ii) Loading 75 µg of lysate and probing the western blot with a 1:500 LC3 antibody dilution could detect both LC3 I and II forms. ← GFP-LC3 I, ˂ GFP-LC3 II, →LC3 I, **→** LC3 II.

**Figure S2: Functional depletion of MGRN1 affects TSG101 ubiquitination.**

HeLa cells transiently co-transfected with HA-Ub and GFP-TSG101 constructs along with MGRN1 or MGRN1ΔR were lysed and immunoprecipitated with anti-GFP antibody. *In vivo* ubiquitination was detected by immunoblotting with anti-Ub antibody. Ubiquitination detected in the presence of MGRN1 is severely compromised when MGRN1ΔR is present. This reiterates previously published data to show that enzymatically active MGRN1 is required for TSG101 ubiquitination. In a similar experiment, cells were co-transfected with HA-Ub and GFP-TSG101 along with PrP or PrP(A117V), lysed, immunoprecipitated and analyzed for TSG101 ubiquitination. Note that the ubiquitination pattern seen in the presence of PrP is similar to that of MGRN1, while PrP(A117V) phenocopies MGRN1ΔR. The input levels of PrP, MGRN1and TSG101 in the total lysates serve as loading control. From this experiment it may be inferred that expression of ^Ctm^PrP severely affects TSG101 ubiquitination, also reaffirming functional depletion of MGRN1 in the presence of enhanced levels of ^Ctm^PrP.

**Figure S3: Depletion of MGRN1 affects exogenously expressed LC3 vesicle processing in a cell line independent manner.**

(A) HeLa cells treated with indicated siRNAs were transfected with RFP-LC3 and imaged.

(B) Graph shows increase in average number of red fluorescent vesicles per cell when MGRN1 is functionally inactive -- MGRN1 knockdown cells have ~16.1 while controls have ~5.5 vesicles per cell. Number of cells analysed, n=40. *** p ≤ 0.001, using Student’s t-test. Error bars, +SEM.

(C) SHSY5Y cells treated with indicated siRNAs were transfected with RFP-LC3 and imaged.

(D) Histogram shows minor but significant increase in size of red vesicles in the presence of MGRN1 siRNA, with an average diameter of ~0.56 µm. In the control cells, the average diameter of vesicles is ~0.48 µm. 204 vesicles were analysed from 15 different cells for each condition. *** p ≤ 0.001, using Student’s t-test. Error bars, +SEM.

(E) Mouse embryonic fibroblast cells (MEFs) were treated with indicated siRNAs, transfected with RFP-LC3 and imaged.

(F) Quantification of this data shows that like cell lines, even in primary cells, MGRN1 depletion results in a marginal increase in size of RFP-LC3 vesicles with average diameter of ~0.37 µm compared with the control (~0.32 µm). 80 vesicles were analysed for each data set. Error bars, +SEM.

(G) HeLa cells transiently co-transfected with MGRN1 or MGRN1ΔR and mCherry-EGFP-LC3B were imaged. Note that expression of MGRN1ΔR closely phenocopies MGRN1 knockdown.

(H) Graph represents ~2.5 folds decrease in the percentage of red vesicles in the presence of MGRN1ΔR compared to MGRN1 controls. Data for panel G was analysed for this graph. 55 cells were analyzed. *** p ≤ 0.001, using Student’s t-test. Error bars, +SEM.

(I) SHSY5Y cells treated with indicated siRNAs were transfected with mCherry-EGFP-LC3B construct. 72 hours post-transfection, cells were fixed and imaged.

(J) Graphical presentation showed ~2.0 folds reduction in the percentage of acidic red vesicles upon expression of MGRN1 siRNA as compared with the control. Data for panel I was analysed for this graph. It shows comparable results between SHSY5Y and HeLa cells. 26 cells were analyzed.

(K) SHSY5Y cells transiently co-transfected with MGRN1 or MGRN1ΔR and mCherry-EGFP-LC3B and treated with 10µM all-*trans*-retinoic acid for 4 day to differentiate. Cells were then imaged. Scale bar, 10μm. The immunoblot on the right shows expression of MGRN1 and MGRN1ΔR.

(L) Additional representative images of cells as in Figure 2G. Expression of MGRN1ΔR in melan a6 cells affects autophagosomal-lysosomal fusion, on the contrary, exogenous expression of MGRN1 in melan md1-nc cells partially compensates for the absence of the protein in these cells. Scale bar, 5μm. *** p≤0.001, using Student’s t-test. Error bars, +SEM. Scale bar, 5μm.

(M) HeLa cells transiently co-transfected with MGRN1 or MGRN1ΔR and mCherry-EGFP-LC3B and treated with vehicle (DMSO) or bafilomycin A1 and imaged. Scale bar, 5μm.

(N) HeLa cells transiently co-transfected with WT PrP or PrP(A117V) and mCherry-EGFP-LC3B and treated with DMSO (vehicle) or bafilomycin A1 and imaged. Scale bar, 5μm.

**Figure S4: Time-lapse images to follow maturation of mCherry-EGFP-LC3B vesicles.**

(A) HeLa cells treated with mock siRNAs were transfected with mCherry-EGFP-LC3B and live cells were imaged. This figure shows images of the entire cell that was followed over the indicated period of time. Inset demarcates the vesicle which was tracked in real time as shown in Figure 3.

(B) Similar experiment monitoring a single live cell over time as in panel A, except for siRNA mediated depletion of MGRN1 in these cells.

(C) Additional examples for Figure 3, where HeLa cells treated with MGRN1 siRNAs were transfected with mCherry-EGFP-LC3B and live cells were imaged. Images of the entire cell are shown over the indicated period of time. Scale bar, 5μm

(D) Inset demarcates the vesicle which was tracked in real time.

**Figure S5: MGRN1 affects autophagic flux.**

(A) HeLa cells were transiently co-transfected with MGRN1 (as control) or MGRN1∆R. These were either treated with 300nM bafilomycin A1 or left untreated, lysed and immunoblotted to check LC3 I and LC3 II. Note that LC3 I is detected only with higher exposures. The levels of GAPDH serve as loading control. Expression of MGRN1 and MGRN1∆R was checked.

(B) HeLa cells were transiently co-transfected with MGRN1 (as control) or MGRN1∆R and GFP-LC3. These were either treated with 300nM bafilomycin A1 or left untreated, lysed and immunoblotted to check for the indicated proteins. Note faint and dark exposures of the blot against GFP show negligible amounts of GFP-LC3 II in control cells without drug treatment. The blots are representative of at least 3 experiments. The levels of GAPDH serve as loading control. Expression of MGRN1 and MGRN1∆R was checked.

(C) Quantification of data from blots produced in panel B shows fold change of endogenous LC3 II level when normalized against GAPDH from 3 independent experiments. ** p≤ 0.05, n.s not significant (p = 0.63), using Student’s t-test. Error bars, +SEM.

(D) SHSY5Y transiently expressing MGRN1 or MGRN1∆R were either treated with 60nM bafilomycin A1 or left untreated; these were lysed and immunoblotted to check for the indicated proteins. The blots are representative of at least 3 experiments. The levels of GAPDH serve as loading control. Expression of MGRN1 and MGRN1∆R was checked.

(E) Quantification of data from panel D denotes fold change in endogenous LC3 II level when normalized against GAPDH from 3 independent experiments. ** p≤ 0.05, n.s. not significant (p = 0.5), using Student’s t-test. Error bars, +SEM.

(F) melan a-6 treated with the indicated siRNAs, lysed and immunoblotted to check for the levels of endogenous LC3 II and p62 in the presence or absence of 50nM bafilomycin A1. The levels of β–tubulin serve as loading control. Note increase in LC3 II levels after depletion of MGRN1 even in melan a-6 cells. Efficiency of knockdown was shown using anti-MGRN1 antibody. This is representative of two independent experiments.

(G) Quantification of data from panel F denotes fold change in endogenous LC3 II level when normalized against β–tubulin.

(H) UbG76V-GFP transfected cells were either left untreated or treated with 300nM bafilomycin A1 or 10μM MG132. Samples were probed with GFP, p62 and β-catenin antibodies. Ponceau-S stained membrane was used as loading control.

**Figure S6: Functional depletion of MGRN1 perturbs the endo-lysosomal pathway, its fusion with the lysosomes and subsequent clearance of cargo.**

(A) HeLa cells treated with irrelevant (GFP) or MGRN1 siRNAs were subjected to Alexa-Fluor 488 EGF uptake. Cells were then washed, fixed at indicated time points and imaged to monitor the fate of the fluorescent EGF-EGFR complex. Scale bar, 5μm.

(B) Cells were similarly treated as in panel A, except untagged EGF was used to stimulate EGFR-mediated uptake, trafficking and degradation. β-tubulin was used as loading control. Efficiency of knockdown was confirmed by immunoblotting with anti-MGRN1. The blots are representative of at least 3 experiments.

**Figure S7: Lysosomal pH measurement using confocal microscopy.**

(A) pH calibration curve for the LysoSensor yellow/blue DND-160–Dextran indicator dye. The 530/440nm fluorescence ratio (R/G) of dextran loaded vesicles was measured.

(B) Histogram showing the average pH of lysosomes in HeLa cells treated with irrelevant (GFP) or MGRN1 siRNAs, as analyzed from around 100 vesicles in the acidic pH range (4.0-5.5). Average lysosomal pH in control and MGRN1 siRNA treated cells were 4.71+0.04 and 4.77+0.03, respectively. n.s non significant (p value=0.9), using Student’s t-test. Error bar, +SEM.

(C) Confocal images representating lysosomal pH as indicated by LysoSensor yellow/blue DND-160–Dextran indicator dye in HeLa cells treated with irrelevant (GFP) or MGRN1 siRNA. The emission at 530 and 440 nm were assigned magenta and blue colors, respectively.
